# Understanding the role of cognitive constructs employed in reading in global math and science achievement

**DOI:** 10.3389/fpsyg.2024.1470977

**Published:** 2024-11-25

**Authors:** Gökhan Arastaman, Metin Bulus, Hakkı Kontaş, Bahadır Özcan

**Affiliations:** ^1^Department of Educational Sciences, Faculty of Education, Hacettepe University, Ankara, Türkiye; ^2^Department of Educational Sciences, Faculty of Education, Adiyaman University, Adıyaman, Türkiye; ^3^Department of Child Development, Anamur Vocational School, Mersin University, Mersin, Türkiye

**Keywords:** reading literacy, math literacy, science literacy, cognition, academic performance, gender, socioeconomic status, PISA

## Abstract

We utilized PISA-2018 data from 71 countries to investigate the relationship between cognitive constructs employed in reading literacy (locating information, understanding, evaluating and reflecting, single and multiple thinking) and math and science achievement. We found that these cognitive constructs collectively accounted for 56% of the variance in math and 63% in science achievement, even after adjusting for gender, socioeconomic status, and country fixed effects. This means that the majority of cultural differences in math and science achievement (resulting from different education systems) can be explained by cognitive constructs employed in reading. We also noted that, at the country level, coefficients of cognitive constructs employed in reading demonstrated an interesting reconciliatory pattern. Countries with a weaker coefficient on the “locating information” dimension tended to have stronger coefficients on the “understanding” and “evaluation and reflection” dimensions; whereas, countries with a stronger coefficient on “understanding” dimension tended to have a weaker coefficient on “evaluation and reflection” dimension. These findings are particularly significant for STEM interventions aiming to enhance math and science achievement, as they indicate that a substantial portion of the variance in these achievements can be explained by cognitive constructs employed in reading literacy. Furthermore, culture-specific reconciliatory patterns imply that strengths in certain cognitive skills can compensate for weaknesses of others. Therefore, schools should consider modifying their curricula to integrate cognitive constructs employed in reading literacy more into math and science education.

## Introduction

The topic of which factors affect academic performance is a long-standing debate in educational research (Guez et al., 2018). For decades, understanding academic achievement and its determinants has attracted considerable attention from researchers and practitioners. Numerous studies collect and analyze data on various factors that affect learning (O'Connell and Marks, 2022). This wealth of evidence has prompted multiple attempts to develop theories of academic achievement (Bloom, 1976; Carroll, 1963; Glaser, 1980). One of the most prominent factors determining academic achievement is the cognitive capacities of individuals. Cognition has a noteworthy impact on understanding, analyzing, and organizing the learning process. The cognitive capacities of individuals in different fields such as mathematics and science literacy can play a decisive role in academic performance (Deary et al., 2007; Roth et al., 2015). However, the complex relationships of these variables have not been comprehensively investigated in different samples. Moreover, the most frequently repeated determinants of learning outcomes are cognitive capacities, SES, and gender (Boman, 2023; Lee and Borgonovi, 2022; Marks and O'Connell, 2021).

Cognitive ability affects academic achievement (Deary et al., 2007; Shi and Qu, 2022; Sternberg et al., 2008). There is broad agreement regarding the positive relationship between cognitive ability and academic achievement (Chen et al., 2012). Studies have shown that academic achievement is related to basic cognitive processes (Tikhomirova et al., 2020). Cognition predicts academic achievement from elementary school through graduate school (Greene et al., 2018). Moreover, academic performance and cognitive abilities predict each other in the development process (Peng and Kievit, 2020). Cognitive ability accounts for from 51 to 75% of the variance in academic achievement alone (Rohde and Thompson, 2007). Specifically, later research such as Flores-Mendoza et al. (2021) and Pokropek et al. (2022) confirmed the strong relationships between performances on PISA and general cognitive ability. Therefore, to understand PISA scores, it is appropriate to examine cognitive constructs’ associations with PISA performance at the country level (Boman, 2022). Consequently, the effects of cognitive abilities on academic performance have been frequently investigated. The current study is different from previous research in addressing the relationship between cognitive constructs employed in reading texts and math and science performance. In addition, these relationships were analyzed for 71 countries overall and at each country level, which is another significance of the research.

Science, mathematics, and reading literacy are some of the most basic required skills in contemporary societies. Individuals’ capability to understand scientific and mathematical concepts, develop critical thinking skills, and solve complex problems is indispensable to adapt and succeed in today’s complex world (McConney et al., 2014; Zan and Di Martino, 2007). Science, mathematics, and reading literacy is a factor that affects not only personal lives but also societies and economies (Bybee and McCrae, 2011). Therefore, it is of great importance to understand the relationship between cognition and academic performance to develop science, mathematics, and reading literacy.

Besides cognitive capacities, socioeconomic status is an important factor influencing academic performance (Bradley and Corwyn, 2002; Davis-Kean, 2005). Indeed, socioeconomic status, family income, parent’s education level, and living environment can affect individuals’ access to educational resources and benefit from them for academic achievement (Hattie, 2009; Sirin, 2005). The impact of socioeconomic status on academic performance needs to be clarified across different countries. Furthermore, gender is an important variable affecting academic performance. Large-scale international or country-level investigations have shown trivial gender differences in this regard (Else-Quest et al., 2010; OECD, 2010). Previous studies have shown that gender may play a role in explaining differences in academic performance, but the causes and effects of these differences are still a matter of debate (Fryer and Levitt, 2010; Kenney-Benson et al., 2006; Lindberg et al., 2010).

### The purpose of the study

Cognitive constructs include mental activities such as thinking, problem-solving, analyzing, critical thinking, and learning. These cognitive constructs considerably affect students’ ability to understand science, mathematics, and reading. Students with well-developed cognitive constructs understand abstract concepts faster, solve complex problems, and learn information more deeply. However, the complex relationships of these variables are relatively new and have not been fully investigated in different samples. Assessing the relationship between cognitive constructs and academic performance among countries participating in the PISA would help us understand the effects of different education systems. This analysis can serve as an example for other countries, highlighting teaching approaches and student support systems in high-achieving countries. It can also shed light on areas for improvement by identifying weak spots in under-performing countries. This research would provide important insights by examining the relationship between cognitive constructs employed in reading and academic performance across the countries participating in PISA 2018. The results of this study can offer important suggestions for shaping education policies, improving teaching methods, and increasing student academic performance.

**Figure 1:**
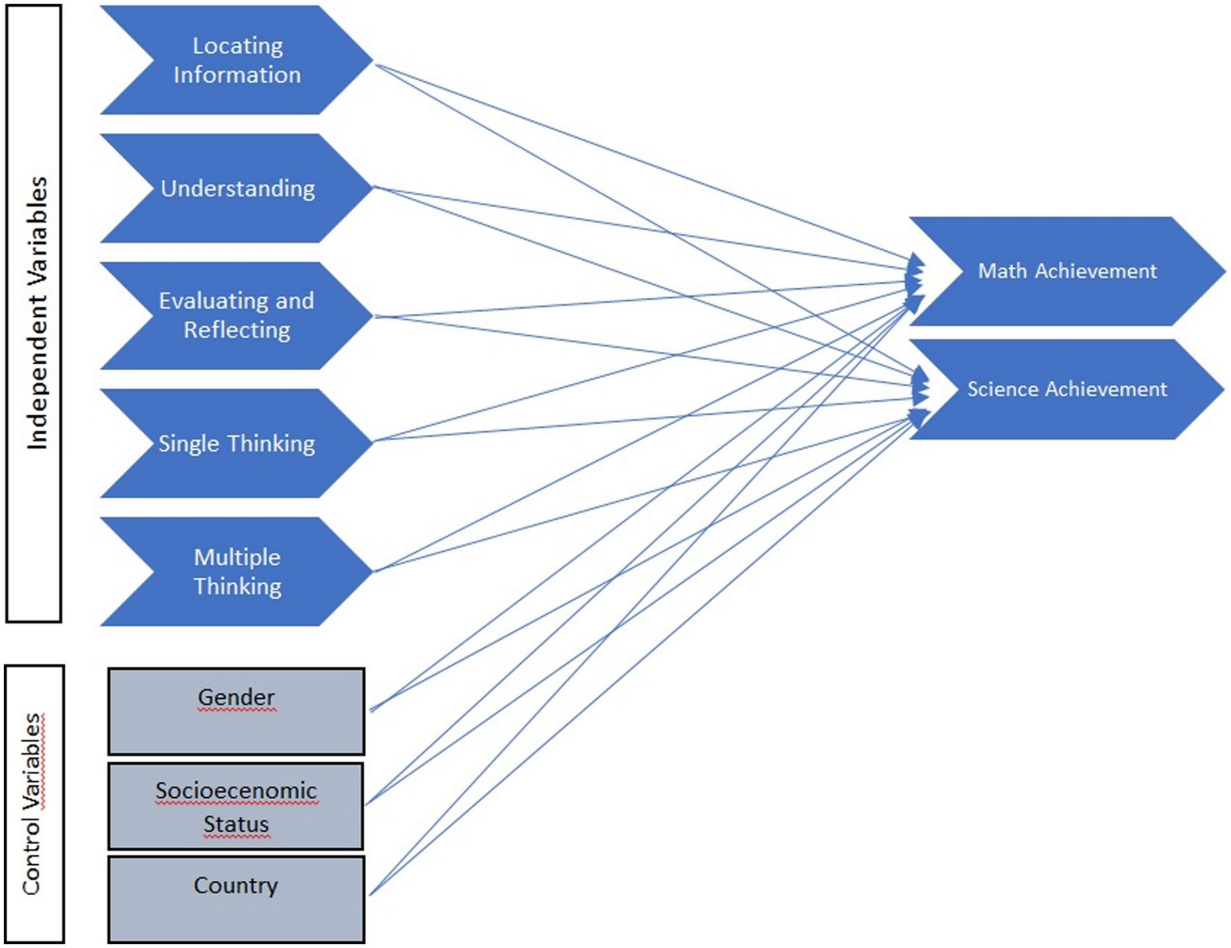
A diagram of the model on cognitive constructs (locating information, understanding, evaluating and reflecting, single and multiple thinking), and math and science achievement.

We proposed the model in which cognitive constructs (locating information, understanding, evaluating and reflecting, single and multiple thinking) employed in reading would significantly relate to math and science achievement after controlling gender, socioeconomic status, and country in the current study (see Figure 1). It aimed to explore the relationships of cognitive constructs (locating information, understanding, evaluating and reflecting, single and multiple thinking) with science and mathematics literacy across 71 countries participating in the PISA 2018 assessment. More specifically, we aim to explore the following research questions:

1. How do cognitive constructs employed in reading literacy relate to science and math achievement after controlling for gender and socioeconomic status?
2. How consistent are these relationships across different education systems?
3. Do education systems explain additional variation in mathematics and science achievement, above and beyond cognitive constructs?

### Theoretical framework

In recent years, large-scale exams have been used to evaluate student performance at national and international levels. International comparisons can be conducted by administering comprehensive exams in multiple countries to gather important details about education systems. One of the most comprehensive large-scale exams in the world is the Program for International Student Assessment (PISA). In addition to assessing 15-year-old students’ science, mathematics, and reading skills, PISA has measured cognitive skills such as locating information, understanding, evaluating and reflecting, single thinking, and multiple thinking employed in reading literacy across PISA participants countries (OECD, 2019c).

PISA test results provide in-depth information about participating countries, the effectiveness of different education systems, the impact of teaching methods, and the causes of student achievement (OECD, 2019c). Measuring students’ cognitive processes such as finding, understanding, evaluating information, and single and multiple thinking employed in reading literacy with PISA data offers a comprehensive perspective to evaluate and improve education systems (Schleicher, 2019). In this context, the impact of cognitive constructs on students’ performance in science and mathematics literacy is important in shaping educational policies (Hopfenbeck et al., 2018; Zhang and Bae, 2020). The current study’s findings would provide a scientific basis to strengthen education systems and enhance the development of student’s cognitive skills. It would shed light on strategies to improve the quality of education on a global scale by revealing the differences and similarities between cognitive constructs and academic performances across participating countries of PISA 2018. Thus, understanding the relationships between students’ cognitive constructs and academic achievement might form the basis of effective policy formulation and implementation for each of the PISA participant countries.

Culture shapes human cognition, and as a result of this cognitive constructs differ from culture to culture (Nisbett, 2004; Markus and Kitayama, 1991). Cultural factors significantly shape cognitive structures and affect how students interact with information, think critically, and reflect on their knowledge, affecting academic achievement (Cole and Packer, 2019; Liu and Nesbit, 2024). Different cultural values and educational practices can lead to various cognitive styles, learning orientations, and information-processing methods (Scholes, 2020). For example, in Western countries such as the US and the UK, education often focuses on critical thinking, individual analysis, and open discussion (Meng et al., 2016). Students are encouraged to question, evaluate, and reflect on information, thus promoting a multi-perspective thinking approach. This educational style develops higher levels of metacognition and supports their ability to evaluate and reflect on information independently (Wu et al., 2020). Educational approaches in many Asian cultures (e.g., China, and Japan) traditionally emphasize memorization, structure, and teacher-led instruction, prioritizing harmony and collective knowledge over individual interpretation. This approach reinforces single-perspective thinking and retention of certain knowledge as students focus on learning established knowledge before developing personal interpretations (Cho et al., 2023). On the other hand, the education system in Scandinavian countries (e.g., Finland) is known for its emphasis on collaboration, hands-on learning, and problem-solving. This student-centered approach encourages both single- and multi-perspective thinking, allowing students to understand key concepts before exploring alternative views and encouraging reflection and evaluation of information as part of collaborative work (Scholes, 2020).

### The relationship between cognitive processes, academic performance and SES

Cognitive capability is a general mental ability that includes reasoning, problem-solving, planning, abstract thinking, comprehending complex ideas, and learning from experiences (Gottfredson, 1997). While cognitive abilities are often measured by short or comprehensive IQ tests, PISA measures a range of cognitive skills in three domains: mathematics, science, and reading and their relationship to real-life situations and contexts in the 21st century (OECD, 2019c). PISA attempts to measure students’ different skills to cope with the cognitive challenges of modernity (the knowledge and skills required for full participation in the knowledge society, overcoming real-life challenges) (OECD, 2010). PISA does not measure pure academic knowledge but focuses on the measurement of competencies that can be attained academically outside school (OECD, 2019c; Rindermann and Baumeister, 2015).

Cognitive constructs used in this study, locating information, understanding, evaluating and reflecting, single and multiple thinking, employed in reading in PISA 2018. Locating information refers to students’ capacity to identify, retrieve, and gather relevant information from texts or data sources (Armbruster and Armstrong, 1993). In the context of PISA, locating information involves navigating quickly through texts or data, finding explicit details, understanding headings, identifying keywords, and distinguishing essential information from non-essential information. This skill is tested by presenting texts, tables or other information formats in which students must find specific data points or answers (Rindermann, 2007). Understanding encompasses students’ ability to grasp the main ideas and underlying meanings in texts and to connect information logically (European Commission: Directorate-General for Education, Youth, Sport and Culture, 2019). Evaluating and reflecting involves students’ ability to critically evaluate information, make judgments, and apply prior knowledge to interpret or critique content. Within the PISA framework, single-thinking tasks focus on students’ ability to understand and apply information from a single source and often require them to follow a direct, single line of reasoning (Teig et al., 2022). On the other hand, multiple thinking tasks challenge students to synthesize and reconcile information across different sources, perspectives, or types of data and encourage them to create comprehensive analyses (Rindermann and Baumeister, 2015).

Students’ socioeconomic status (SES) has been used often to explain academic achievement. Based on the SES model, numerous publications have been produced on the scope of socioeconomic inequalities in education, theoretical explanations and measurement of their relationship to education, and policies aimed at reducing inequalities in education (Marks and O'Connell, 2021). SES and academic achievement relationship has been consistently confirmed in national and international contexts with a significant body of research (Bradley and Corwyn, 2002; Coleman et al., 1966; Davis-Kean, 2005; Hattie, 2009; Sirin, 2005). In addition to explaining academic achievement, SES might be used to decrease the academic achievement gap and social inequality that exists all over the World (Sirin, 2005).

There is a connection between international academic assessment scores and SES across countries (Flores-Mendoza et al., 2021; Rindermann, 2018). SES is a multifaceted concept based on theoretically contradictory assumptions (Avvisati, 2020; Lee and Borgonovi, 2022). Some studies of SES have emphasized the cultural achievements of middle- and upper-class families, while others have focused on the interaction between high SES students and students’ abilities acquired in schools (Milne and Aurini, 2015; Myrberg and Rosén, 2009). Although the effects of SES on academic performance have varied across countries and cultures, SES has been directly or indirectly associated with high academic achievement (Bray, 2006; Lee and Borgonovi, 2022; Sackett et al., 2009; Sirin, 2005). However, Marks and O'Connell (2021) have argued that the exact relationship between SES and academic achievement needs to be clarified through further studies. Since cognitive ability cannot fully explain SES and SES cannot fully explain cognitive ability, these two constructs should be studied in multivariate models (Boman, 2022). Although SES is an important variable at the individual level, it is likely to affect the country-level PISA scores (Burhan et al., 2017; Flores-Mendoza et al., 2021). On this subject, PISA scores have a stronger relationship with cognitive ability scores than middle-income (Boman, 2023; O'Connell and Marks, 2022). Therefore, countries need average general cognitive ability to develop, then economic development enhances the well-being of families, schools, and the country as a whole, which in turn improves cognitive abilities and school achievement to some extent (Komatsu et al., 2019; Rindermann, 2018).

## Method

### Data

Initiated and supported by Organization for Economic Co-operation and Development (OECD), Programme for International Student Assessment (PISA) collects data on students, their parents, teachers, and school principals using a stratified multi-stage sampling that is representative of the 82 participating countries. We used a subset of the PISA 2018 dataset, including variables of interest from 71 participating countries. Eleven countries (Argentina, Cyprus, Jordan, Lebanon, Moldova, Romania, Saudi Arabia, Vietnam, Ukraine, North Macedonia, and Moscow City) were excluded because one or both of the outcome variables were unavailable. Missing data strategies does not make sense for two reasons, (1) countries are not random (not randomly chosen subset of all countries in the world), and (2) the outcome variable is completely missing, making it impossible to impute at the student level. Finally, there were 551,930 students from 19,664 schools across 71 countries. On average, they represent students that were 15 years and 9 months old (SD = 0.29) with a minimum of 15 years and 1 month old and a maximum of 16 years and 4 months old. Half of the students were female.

### Measures

#### Mathematical and scientific literacy

A student adept in mathematics can formulate a mathematical problem based on a real-world context, solve this mathematical problem, interpret results, and evaluate results within the real-world context. PISA 2018 defines mathematics literacy as an “individual’s capacity to formulate, employ and interpret mathematics in a variety of contexts” and includes “reasoning mathematically and using mathematical concepts, procedures, facts and tools to describe, explain and predict phenomena” (OECD, 2019a). Although mathematics literacy was reported as a single scale in PISA 2018, it consists of four subscales; change and relationships, space and shape, quantity, and uncertainty. Each of the four subscales has 25 items totaling 100 embedded within personal, occupational, societal, and scientific contexts.

PISA 2018 defines scientific literacy as being competent in “Explaining phenomena scientifically,” “Evaluating and designing scientific inquiry,” and “Interpreting data and evidence scientifically” (OECD, 2019a). These are also three subscales representing the scientific competency dimension. In addition, scientific literacy is divided by content areas; physical systems, living systems, and earth and space systems. However, in PISA 2018, a single overall score was reported.

Mathematics and science literacy were minor domains in PISA 2018. Although the mode of delivery was mostly computer-based, they were not adaptive; in other words, computer-based and paper-based implementation included the same trend items. A small number of countries used a paper-based approach for delivering the test. Students’ posterior ability distribution was estimated using item response theory and latent regression from which ten plausible values are drawn. These ten plausible values were transformed to have a mean of 500 and a standard deviation of 100 using linear transformation coefficients in PISA 2012 and 2015, in which mathematics and science were the major domains of interest. Increasing scores indicate more proficiency in math and science.

#### Cognitive processes subscales of reading literacy

The definition of reading literacy in PISA 2018 is “understanding, using, evaluating, reflecting on and engaging with texts in order to achieve one’s goals, to develop one’s knowledge and potential and to participate in society” (OECD, 2019a) and is seen as “the foundation for full participation in the economic, political, communal and cultural life of contemporary society” (OECD, 2019a). There are seven aspects of cognitive processes of reading mapping onto three sub-domains (locating information, understanding, evaluating and reflecting). Understanding sub-domain (named as RCUN in data, tables, and figures) involves the ability to acquire a representation of the literal meaning of texts, integrate them with overarching textual contexts, and generate inferences in terms of relating parts with a whole which helps with the identification of the main idea (OECD, 2019a). Locating information sub-domain (named as RCLI in data, tables, and figures) involves the ability to search many texts and select the relevant ones, and access and retrieve relevant information (e.g., a number in a table) (OECD, 2019a). Evaluating and reflecting sub-domain (named as RCER in data, tables, and figures) involves the ability to assess the quality and credibility of texts (e.g., whether it is accurate or up to date), to reflect on the content and form of the texts to relate it to the personal experience, to detect and resolve contradictory claims in the text (OECD, 2019a).

The three sub-domains are measured along the line of two categories of text structure (single- and multiple-source). A reading task with single-source text (named RTSN in data, tables, and figures) has a single author (or a group of authors). In other words, it has a piece of single bibliographical information. On the contrary, a reading task with multiple-source texts (named RTML in data, tables, and figures) has multiple authors and bibliographical information. Single-source reading tasks are appropriate for assessing the ability to scan and locate information in the text, acquire literal meaning, generate inferences to connect the text with overarching textual context, assess the credibility and quality of the text, and reflect on the content and form of the text (OECD, 2019a). Multiple-source reading tasks are appropriate for assessing the ability to generate inferences based on multiple texts, search and select relevant information in the text, and detect and resolve contradictory information (OECD, 2019a).

Most countries deliver reading items using multi-stage computer-adaptive testing (MS-CAT). This mode of administration allows assessing students’ ability with fewer items and more precision along the line of the ability scale. Students’ posterior ability distribution was estimated using item response theory and latent regression from which 10 plausible values are drawn. These 10 plausible values were transformed to have a mean of 500 and a standard deviation of 100 using linear transformation coefficients in PISA 2009, in which reading was the major domain of interest (as is in PISA 2018). Increasing scores indicate more proficiency in reading.

#### Demographic variables

We included gender and the Index of Economic, Social, and Cultural Status (ESCS) as demographic variables. These two variables are of primary or secondary interest to policymakers, researchers, and practitioners. The outcome gap between females and males and between students coming from well-to-do and not-so-well-to-do families are used to inform policy because they are politically relevant; they are used as covariates that need to be controlled for because they could be potential confounders; and they can be used as moderators because an effect of interest (e.g., regression coefficients or treatment effects) may function differentially depending on gender and socioeconomic status (e.g., Acar-Erdol and Akin-Arikan, 2022; Bulus and Koyuncu, 2021; Cicek et al., 2021; Dong et al., 2022; Koyuncu et al., 2022; Otanga et al., 2021; Ozcan and Bulus, 2022).

The gender variable was recoded, so females were 1 and males were 0 (named FEMALE in data, tables, and figures). The coefficient on the FEMALE variable is the achievement gap between females and males, or to what extent females perform compared to males. The ESCS index was computed as the arithmetic mean of the three standardized variables; parents’ highest occupation (determined based on international standard classification of occupations), parents’ years of education (determined based on international standard classification of education), and home possessions (e.g., books) representing family wealth (OECD, 2019b). If any of the three variables were missing, it was predicted using the other two in the regression model, and some random noise was added to the predicted value. If more than one variable was missing among the three, ESCS was not computed, and a missing value was assigned. Unlike earlier cycles, in PISA 2018, all three variables were standardized based on both OECD and partner countries. Furthermore, in PISA 2018, all three variables contributed to the composite ESCS scale with equal weights of 1, meaning that it can be derived using the simple arithmetic mean of three components. The final composite score was transformed into a weighted (SENWT) OECD mean of zero and a standard deviation of one. Increasing values indicate that students come from well-to-do families.

### Preliminary analysis

We report descriptive statistics for the overall sample (*N* = 551,930) in Table 1. Mean, standard deviation, skewness, and excess kurtosis values were obtained via weighting observations with SENWT (totaling *N* = 355,000). Skewness and excess kurtosis values were within an acceptable range of −2 and +2 (Byrne, 2016; George and Mallery, 2019; Hair et al., 2010). Half of the students were female. Partner countries participating in PISA 2018 drag ESCS to 0.28 standard deviation below the OECD mean of zero. Similarly, they drag proficiency scales to 35–43 points below the OECD average of 500.

**Table 1:** Descriptive statistics for variables of interest (overall sample).

Variable	Mean	SD	Median	Min.	Max.	Skew.	Excess Kurt.	Valid *N*	
								Unweighted	Weighted
Math	465.52	104.72	466.89	25.17	895.30	−0.01	−0.35	551,930	355,000
Science	463.20	103.16	462.71	43.10	883.93	0.09	−0.42	551,930	355,000
Female	0.50	0.50	0.00	0.00	1.00	0.02	−2.00	551,928	355,000
ESCS	−0.28	1.12	−0.14	−8.17	4.21	−0.53	0.21	538,111	346,548
RCLI	458.19	113.97	461.63	2.72	905.05	−0.06	−0.40	551,930	355,000
RCUN	459.04	110.82	460.85	31.66	894.31	0.01	−0.47	551,930	355,000
RCER	460.93	113.63	462.75	7.56	935.82	0.06	−0.46	551,930	355,000
RTSN	457.17	112.09	459.70	18.21	903.05	−0.01	−0.45	551,930	355,000
RTML	461.58	110.88	463.27	33.13	905.21	0.03	−0.47	551,930	355,000

SD, Standard Deviation. Mean, SD, skewness, and excess kurtosis were weighted with SENWT. FEMALE (0 = Male, 1 = Female). ESCS: index of economic, social and cultural status. RCLI: cognitive process subscale of reading - locate information. RCUN: cognitive process subscale of reading–understand. RCER: cognitive process subscale of reading–evaluate and reflect. RTSN: text structure subscale of reading–single. RTML: text structure subscale of reading–multiple.

Bivariate correlations (weighted with SENWT) are reported in Table 2. We interpret the magnitude of correlations in light of Cohen (1988), Gignac and Szodorai (2016), and Lovakov and Agadullina (2021). There is a small correlation between FEMALE and subscales of reading, a moderate to a large correlation between ESCS and subscales of reading, a large correlation between MATH and SCIENCE and subscales of reading, and a very large correlation between subscales of reading. Being female is associated with higher scores on reading subscales (0.11 ≤ *r* ≤ 0.14) but not on math and science. Coming from well-to-do families is associated with higher scores on math, science, and reading subscales (0.40 ≤ *r* ≤ 0.43). Students scoring high on reading subscales also score high on math and science (0.80 ≤ *r* ≤ 0.86). Students scoring high on any of the subscales of reading also score high on other subscales of reading (0.90 ≤ *r* ≤ 0.95). The very large correlation between reading subscales could potentially cause a collinearity problem. We inspected and reported the variance inflation factor (VIF) in Table 2. They are at a margin or lower than the suggested cutoff value of 10 (Kline, 2016). We continued the analysis using the complete set of variables.

**Table 2:** Bivariate correlations and variance inflation factors (Overall Sample).

	(1)	(2)	(3)	(4)	(5)	(6)	(7)	(8)	(9)	VIF_SCIENCE_
(1) Math										*NA*
(2) Science	0.85									*NA*
(3) ESCS	0.43	0.41								1.03
(4) FEMALE	−0.02	0.02	−0.01							1.20
(5) RCLI	0.80	0.84	0.41	0.13						6.46
(6) RCUN	0.81	0.86	0.40	0.13	0.94					10.61
(7) RCER	0.80	0.85	0.42	0.11	0.92	0.95				7.04
(8) RTSN	0.81	0.85	0.41	0.14	0.90	0.92	0.90			7.12
(9) RTML	0.81	0.86	0.41	0.12	0.90	0.92	0.91	0.95		7.41
VIF_MATH_	*NA*	*NA*	1.03	1.24	6.34	9.99	6.40	6.24	6.41	

VIF, variance inflation factor. Observations were weighted with SENWT. FEMALE (0 = Male, 1 = Female). ESCS: index of economic, social and cultural status. RCLI: cognitive process subscale of reading–locate information. RCUN: cognitive process subscale of reading–understand. RCER: cognitive process subscale of reading–evaluate and reflect. RTSN: text structure subscale of reading–single. RTML: text structure subscale of reading–multiple.

### Analytic strategy

The study analyzed data from the PISA 2018 survey. Descriptive statistics like means, standard deviations, skew and kurtosis, and correlations were weighted using the survey weights (SENWT) to ensure equal contribution by each country. SENWT was also used for by-country estimation to ensure comparability of estimates. Outcome variables had 10 plausible values, so descriptive statistics were averaged across these values. We used a restricted maximum likelihood estimator in Mplus and forced all countries to have the same model.

## Results

The result of multi-group path analysis by country and subject, overall sample, and overall sample with country fixed effects are reported in Table 3. The values in the table are standardized regression coefficients, their standard errors (in parenthesis), the total $R^{2}$ value for the full model (all variables in the table), and the $\Delta R^{2}$, which is the unique contribution of the subscales of reading compared to the demographic-only model (FEMALE and ESCS). Although results from the overall sample and the overall sample with country fixed effects are similar, we report results of the overall sample with country fixed effects. Considering the contextual effect of countries, females scored substantially lower on math and science despite controlling for socioeconomic status and reading. They scored 0.23 (*p* < 0.001) and 0.18 (*p* < 0.001) standard deviation below males in math and science, which is equivalent to dragging an average student from the 50th percentile to 41st and 43rd percentile, correspondingly (calculated using PowerUpR; Bulus et al., 2021). The benefit of coming from a more well-to-do family was minuscule after controlling for students’ gender and reading ability. A standard deviation increase on the ESCS was associated with a measly 0.07 (*p* < 0.001) standard deviation increase in math and 0.04 (*p* < 0.001) standard deviation increase in science. This is an important finding because what we know about the socioeconomic gap seems to be mainly driven by a lack of resources to prepare students for reading literacy.

**Table 3:** Regression coefficients, standard errors (in parenthesis), and *R*^2^ values by country and subject.

Country	Subject	Female	ESCS	RCLI	RCUN	RCER	RTSN	RTML	*R^2^*	Δ *R^2^*
Albania	Math	−0.29(0.03)***	−0.04(0.02)*	0.23(0.07)***	0.07(0.07)	0.09(0.09)	0.14(0.05)**	0.25(0.06)***	0.51	0.47
	Science	−0.17(0.03)***	0.02(0.01)*	0.13(0.05)**	0.06(0.07)	0.23(0.04)***	0.21(0.05)***	0.22(0.04)***	0.63	0.56
Baku (Azerbaijan)	Math	−0.34(0.03)***	0.06(0.01)***	0.07(0.06)	0.18(0.1)+	0.15(0.07)*	0.26(0.03)***	0.12(0.04)**	0.56	0.51
	Science	−0.20(0.03)***	0.02(0.02)**	0.01(0.04)	0.24(0.07)***	0.17(0.05)***	0.25(0.03)***	0.15(0.04)***	0.59	0.56
Australia	Math	−0.28(0.03)***	0.07(0.01)***	0.09(0.03)**	0.09(0.06)	0.25(0.04)***	0.21(0.05)***	0.20(0.04)***	0.65	0.54
	Science	−0.26(0.02)***	0.03(0.01)***	0.07(0.03)**	0.18(0.04)***	0.20(0.04)***	0.21(0.04)***	0.23(0.03)***	0.74	0.64
Austria	Math	−0.37(0.03)***	0.07(0.01)***	0.12(0.04)**	0.15(0.08)+	0.18(0.07)*	0.29(0.04)***	0.12(0.04)**	0.73	0.58
	Science	−0.25(0.02)***	0.06(0.01)***	0.08(0.04)**	0.16(0.05)+	0.23(0.04)***	0.27(0.05)***	0.16(0.04)***	0.78	0.63
Belgium	Math	−0.29(0.03)***	0.1(0.01)***	0.08(0.04)+	0.19(0.06)**	0.18(0.04)***	0.25(0.04)***	0.14(0.04)**	0.73	0.52
	Science	−0.23(0.02)***	0.07(0.01)***	0.05(0.04)+	0.21(0.03)***	0.21(0.03)***	0.26(0.04)***	0.16(0.03)***	0.79	0.59
Bosnia and Herzegovina	Math	−0.31(0.03)***	0.06(0.01)***	0.26(0.05)***	0.06(0.07)	0.10(0.06)	0.15(0.04)***	0.25(0.05)***	0.63	0.55
	Science	−0.29(0.02)***	0.03(0.01)**	0.24(0.06)***	0.07(0.07)	0.12(0.03)***	0.19(0.05)***	0.24(0.05)***	0.67	0.60
Brazil	Math	−0.29(0.02)***	0.10(0.02)***	0.14(0.04)***	0.12(0.04)**	0.15(0.04)***	0.24(0.05)***	0.17(0.05)**	0.67	0.51
	Science	−0.19(0.02)***	0.08(0.01)***	0.09(0.04)**	0.20(0.04)***	0.15(0.03)***	0.29(0.04)***	0.13(0.03)***	0.73	0.57
Brunei Darussalam	Math	−0.17(0.02)***	0.02(0.01)	0.17(0.04)***	0.20(0.05)***	0.08(0.06)	0.28(0.06)***	0.17(0.05)**	0.77	0.63
	Science	−0.19(0.03)***	0.02(0.01)	0.09(0.04)**	0.22(0.05)***	0.15(0.04)***	0.30(0.06)***	0.18(0.05)***	0.82	0.67
Bulgaria	Math	−0.28(0.03)***	0.07(0.01)***	0.15(0.05)**	0.00(0.08)	0.26(0.07)***	0.21(0.06)***	0.18(0.05)***	0.63	0.49
	Science	−0.16(0.02)***	0.06(0.01)***	0.04(0.04)**	0.14(0.06)	0.27(0.05)***	0.21(0.05)***	0.22(0.05)***	0.75	0.58
Belarus	Math	−0.28(0.02)***	0.07(0.01)***	0.12(0.07)+	0.14(0.07)*	0.16(0.06)**	0.23(0.04)***	0.21(0.04)***	0.73	0.53
	Science	−0.26(0.02)***	0.01(0.01)**	0.04(0.05)+	0.23(0.05)***	0.19(0.05)***	0.23(0.04)***	0.22(0.03)***	0.76	0.59
Canada	Math	−0.27(0.02)***	0.08(0.01)***	0.06(0.05)	0.15(0.04)**	0.19(0.04)***	0.12(0.06)*	0.26(0.04)***	0.57	0.49
	Science	−0.20(0.03)***	0.03(0.01)**	0.02(0.03)	0.22(0.04)***	0.2(0.03)***	0.16(0.03)***	0.27(0.02)***	0.69	0.62
Chile	Math	−0.23(0.02)***	0.13(0.02)***	0.13(0.04)**	0.08(0.06)	0.18(0.06)**	0.21(0.05)***	0.16(0.05)***	0.62	0.45
	Science	−0.19(0.02)***	0.08(0.01)***	0.07(0.05)**	0.17(0.05)***	0.18(0.05)***	0.16(0.03)***	0.25(0.04)***	0.69	0.54
Chinese Taipei	Math	−0.21(0.03)***	0.07(0.02)***	0.05(0.06)	0.22(0.07)**	0.14(0.06)*	0.29(0.06)***	0.15(0.05)**	0.70	0.57
	Science	−0.20(0.02)***	0.03(0.01)**	0.06(0.04)	0.26(0.06)***	0.12(0.06)*	0.35(0.04)***	0.11(0.04)**	0.77	0.66
Columbia	Math	−0.31(0.03)***	0.05(0.02)*	0.09(0.05)+	0.18(0.07)*	0.12(0.08)	0.23(0.07)**	0.18(0.04)***	0.63	0.50
	Science	−0.24(0.02)***	0.00(0.01)*	0.10(0.04)+	0.17(0.06)*	0.17(0.04)***	0.27(0.05)***	0.16(0.04)***	0.71	0.59
Costa Rica	Math	−0.34(0.04)***	0.06(0.02)**	0.12(0.07)	0.06(0.07)	0.25(0.04)***	0.22(0.09)*	0.15(0.09)	0.61	0.46
	Science	−0.24(0.03)***	0.08(0.01)***	0.09(0.07)	0.13(0.08)	0.21(0.03)***	0.22(0.05)***	0.19(0.05)***	0.70	0.52
Croatia	Math	−0.38(0.03)***	0.06(0.01)***	0.12(0.06)*	0.11(0.09)	0.22(0.06)**	0.19(0.05)***	0.22(0.05)***	0.68	0.58
	Science	−0.25(0.02)***	0.04(0.01)**	0.03(0.04)*	0.13(0.08)	0.28(0.06)***	0.18(0.04)***	0.25(0.04)***	0.71	0.62
Czech Republic	Math	−0.30(0.03)***	0.09(0.01)***	0.06(0.05)	0.12(0.06)*	0.22(0.06)***	0.25(0.07)***	0.16(0.06)*	0.68	0.49
	Science	−0.26(0.03)***	0.04(0.02)**	0.03(0.06)	0.13(0.07)*	0.27(0.05)***	0.23(0.04)***	0.21(0.04)***	0.74	0.57
Denmark	Math	−0.30(0.03)***	0.06(0.01)***	0.04(0.05)	0.18(0.07)**	0.20(0.06)**	0.20(0.06)**	0.21(0.05)***	0.65	0.54
	Science	−0.24(0.02)***	0.07(0.01)***	−0.01(0.04)	0.26(0.04)***	0.17(0.04)***	0.16(0.06)**	0.29(0.05)***	0.73	0.61
Dominican Republic	Math	−0.24(0.04)***	0.06(0.02)**	0.08(0.06)	0.24(0.08)**	0.08(0.06)	0.27(0.07)***	0.14(0.08)+	0.62	0.52
	Science	−0.16(0.03)***	0.06(0.02)**	0.13(0.05)	0.21(0.07)**	0.10(0.03)	0.20(0.05)***	0.21(0.04)***	0.68	0.57
Estonia	Math	−0.37(0.03)***	0.07(0.01)***	0.05(0.06)	0.16(0.1)	0.21(0.07)**	0.32(0.05)***	0.10(0.05)+	0.65	0.56
	Science	−0.23(0.02)***	0.03(0.01)**	0.06(0.04)	0.18(0.07)	0.22(0.05)***	0.23(0.05)***	0.22(0.05)***	0.74	0.67
Finland	Math	−0.34(0.03)***	0.09(0.01)***	0.07(0.06)	0.18(0.09)+	0.17(0.07)*	0.18(0.05)**	0.24(0.05)***	0.65	0.54
	Science	−0.20(0.03)***	0.05(0.01)***	0.06(0.04)	0.16(0.06)+	0.23(0.05)***	0.22(0.04)***	0.23(0.03)***	0.75	0.63
France	Math	−0.27(0.02)***	0.11(0.01)***	0.14(0.05)**	0.04(0.05)	0.24(0.05)***	0.18(0.04)***	0.24(0.04)***	0.73	0.51
	Science	−0.20(0.02)***	0.08(0.01)***	0.06(0.04)**	0.16(0.05)	0.22(0.04)***	0.24(0.05)***	0.20(0.04)***	0.77	0.57
Georgia	Math	−0.28(0.03)***	0.07(0.01)***	0.11(0.06)+	0.23(0.07)**	0.06(0.06)	0.23(0.06)***	0.17(0.05)**	0.61	0.50
	Science	−0.18(0.03)***	0.05(0.02)**	0.02(0.05)+	0.26(0.06)***	0.14(0.04)	0.21(0.05)***	0.22(0.04)***	0.66	0.55
Germany	Math	−0.30(0.03)***	0.08(0.02)***	0.14(0.07)*	0.11(0.06)+	0.19(0.11)+	0.20(0.04)***	0.21(0.05)***	0.72	0.53
	Science	−0.22(0.02)***	0.07(0.01)***	0.07(0.06)*	0.20(0.07)+	0.19(0.05)***	0.24(0.04)***	0.20(0.04)***	0.78	0.60
Greece	Math	−0.34(0.03)***	0.08(0.01)***	0.11(0.07)	0.12(0.08)	0.17(0.05)***	0.17(0.06)**	0.22(0.07)**	0.62	0.49
	Science	−0.23(0.02)***	0.04(0.01)**	0.10(0.07)	0.14(0.07)	0.19(0.06)**	0.22(0.05)***	0.22(0.04)***	0.71	0.60
Hong Kong	Math	−0.24(0.02)***	0.05(0.02)**	0.07(0.06)	0.13(0.07)+	0.24(0.05)***	0.23(0.04)***	0.17(0.04)***	0.64	0.59
	Science	−0.21(0.03)***	0.05(0.01)***	−0.02(0.05)	0.19(0.06)+	0.26(0.04)***	0.21(0.04)***	0.22(0.05)***	0.70	0.64
Hungary	Math	−0.29(0.03)***	0.1(0.02)***	0.1(0.05)*	0.12(0.06)*	0.21(0.05)***	0.20(0.04)***	0.22(0.04)***	0.74	0.50
	Science	−0.27(0.02)***	0.05(0.02)**	0.09(0.05)*	0.15(0.05)*	0.21(0.05)***	0.19(0.04)***	0.25(0.03)***	0.78	0.57
Iceland	Math	−0.18(0.03)***	0.08(0.02)***	0.01(0.07)	0.15(0.09)+	0.27(0.07)***	0.18(0.09)*	0.19(0.08)*	0.62	0.53
	Science	−0.23(0.03)***	0.06(0.01)***	−0.04(0.04)	0.25(0.07)+	0.23(0.06)***	0.24(0.08)*	0.20(0.06)*	0.75	0.66
Indonesia	Math	−0.11(0.03)**	0.05(0.02)*	0.11(0.04)*	0.23(0.05)***	0.08(0.05)	0.18(0.05)***	0.20(0.05)***	0.58	0.51
	Science	−0.14(0.03)***	0.04(0.02)*	0.20(0.04)***	0.12(0.05)**	0.11(0.04)	0.23(0.05)***	0.18(0.04)***	0.63	0.55
Ireland	Math	−0.28(0.03)***	0.07(0.01)***	0.09(0.05)+	0.10(0.08)	0.22(0.07)**	0.26(0.06)***	0.16(0.05)**	0.68	0.56
	Science	−0.20(0.02)***	0.04(0.01)***	0.07(0.04)+	0.15(0.07)	0.23(0.06)***	0.25(0.04)***	0.20(0.04)***	0.75	0.64
Israel	Math	−0.24(0.03)***	0.08(0.02)***	0.15(0.05)**	0.08(0.05)	0.19(0.06)**	0.23(0.05)***	0.18(0.05)**	0.68	0.53
	Science	−0.17(0.02)***	0.03(0.01)**	0.11(0.04)**	0.15(0.06)	0.19(0.06)**	0.23(0.03)***	0.22(0.04)***	0.76	0.63
Italy	Math	−0.36(0.03)***	0.07(0.02)***	0.15(0.05)**	0.07(0.05)	0.20(0.05)***	0.18(0.05)***	0.23(0.05)***	0.66	0.55
	Science	−0.24(0.02)***	0.02(0.01)**	0.11(0.03)***	0.12(0.04)	0.23(0.04)***	0.23(0.05)***	0.2(0.04)***	0.72	0.63
Kosovo	Math	−0.32(0.03)***	0.02(0.01)+	0.16(0.06)**	0.17(0.06)**	0.11(0.05)*	0.27(0.05)***	0.13(0.04)**	0.61	0.56
	Science	−0.18(0.02)***	0.03(0.01)+	0.14(0.06)**	0.13(0.07)**	0.17(0.05)*	0.31(0.04)***	0.13(0.04)**	0.68	0.62
Japan	Math	−0.27(0.02)***	0.07(0.02)***	0.15(0.05)**	0.07(0.08)	0.19(0.05)***	0.23(0.05)***	0.18(0.04)***	0.65	0.56
	Science	−0.20(0.02)***	0.02(0.01)**	0.13(0.04)***	0.14(0.05)	0.19(0.04)***	0.24(0.04)***	0.21(0.04)***	0.75	0.67
Kazakhstan	Math	−0.25(0.03)***	0.01(0.02)	0.08(0.04)*	0.19(0.04)***	0.10(0.04)**	0.25(0.05)***	0.12(0.05)*	0.46	0.43
	Science	−0.17(0.03)***	0.01(0.01)	0.09(0.03)*	0.24(0.04)***	0.13(0.04)**	0.21(0.04)***	0.20(0.05)***	0.65	0.62
Korea	Math	−0.21(0.03)***	0.1(0.01)***	−0.02(0.05)	0.10(0.06)+	0.34(0.06)***	0.21(0.05)***	0.18(0.06)**	0.65	0.54
	Science	−0.22(0.02)***	0.03(0.01)**	0.01(0.05)	0.10(0.08)+	0.34(0.06)***	0.14(0.04)***	0.28(0.04)***	0.72	0.64
Latvia	Math	−0.36(0.03)***	0.1(0.01)***	−0.01(0.05)	0.14(0.06)*	0.29(0.06)***	0.14(0.06)*	0.24(0.06)***	0.63	0.52
	Science	−0.20(0.03)***	0.04(0.01)**	0.00(0.07)	0.16(0.06)*	0.28(0.05)***	0.2(0.04)***	0.22(0.04)***	0.69	0.60
Lithuania	Math	−0.32(0.03)***	0.06(0.01)***	0.15(0.05)**	0.10(0.05)+	0.18(0.03)***	0.23(0.04)***	0.2(0.06)***	0.69	0.55
	Science	−0.29(0.03)***	0.03(0.01)**	0.11(0.06)**	0.16(0.08)+	0.19(0.06)**	0.17(0.04)***	0.26(0.04)***	0.74	0.61
Luxembourg	Math	−0.27(0.02)***	0.08(0.02)***	0.06(0.06)	0.07(0.07)	0.29(0.07)***	0.22(0.05)***	0.20(0.06)***	0.71	0.52
	Science	−0.16(0.02)***	0.08(0.01)***	0.04(0.04)	0.15(0.07)	0.26(0.05)***	0.24(0.04)***	0.20(0.04)***	0.79	0.58
Macao	Math	−0.23(0.06)***	0.06(0.02)**	0.18(0.06)**	−0.12(0.13)	0.32(0.08)***	0.19(0.09)*	0.19(0.08)*	0.55	0.52
	Science	−0.18(0.03)***	0.01(0.01)**	0.19(0.04)***	0.03(0.08)	0.24(0.05)***	0.15(0.06)*	0.29(0.06)***	0.72	0.70
Malaysia	Math	−0.15(0.03)***	0.08(0.01)***	0.24(0.05)***	0.06(0.05)	0.11(0.05)*	0.21(0.06)***	0.19(0.07)**	0.65	0.48
	Science	−0.17(0.03)***	0.05(0.01)***	0.13(0.03)***	0.18(0.03)***	0.15(0.04)***	0.24(0.05)***	0.20(0.05)***	0.78	0.60
Malta	Math	−0.22(0.03)***	0.11(0.02)***	0.21(0.1)*	0.07(0.12)	0.11(0.1)	0.16(0.09)+	0.29(0.08)**	0.70	0.58
	Science	−0.17(0.03)***	0.07(0.01)***	0.10(0.06)*	0.07(0.1)	0.27(0.07)***	0.29(0.06)***	0.17(0.06)**	0.77	0.66
Mexico	Math	−0.28(0.03)***	0.03(0.02)+	0.14(0.06)*	0.13(0.06)*	0.13(0.07)+	0.32(0.04)***	0.10(0.05)*	0.63	0.51
	Science	−0.25(0.03)***	0.03(0.01)+	0.09(0.05)*	0.18(0.06)*	0.18(0.06)+	0.24(0.04)***	0.18(0.04)***	0.72	0.60
Montenegro	Math	−0.37(0.04)***	0.06(0.02)**	0.15(0.06)**	0.05(0.07)	0.22(0.05)***	0.2(0.05)***	0.20(0.04)***	0.62	0.55
	Science	−0.24(0.02)***	0.02(0.01)**	0.03(0.06)**	0.14(0.07)	0.29(0.04)***	0.21(0.04)***	0.23(0.04)***	0.71	0.65
Morocco	Math	−0.26(0.03)***	0.06(0.02)**	0.14(0.06)*	0.21(0.08)**	0.03(0.08)	0.24(0.04)***	0.16(0.05)***	0.56	0.49
	Science	−0.13(0.03)***	0.01(0.02)**	0.14(0.04)*	0.16(0.07)**	0.13(0.05)	0.28(0.04)***	0.16(0.05)**	0.66	0.59
Netherlands	Math	−0.25(0.02)***	0.07(0.02)***	−0.04(0.05)	0.18(0.06)**	0.35(0.07)***	0.42(0.04)***	−0.06(0.04)+	0.74	0.60
	Science	−0.16(0.02)***	0.06(0.01)***	−0.03(0.05)	0.21(0.06)***	0.35(0.05)***	0.37(0.05)***	−0.01(0.04)+	0.77	0.64
New Zealand	Math	−0.30(0.03)***	0.07(0.01)***	0.09(0.06)	0.09(0.09)	0.23(0.08)**	0.15(0.04)***	0.25(0.05)***	0.65	0.52
	Science	−0.24(0.02)***	0.04(0.01)***	0.04(0.04)	0.17(0.04)***	0.25(0.05)***	0.16(0.03)***	0.28(0.04)***	0.77	0.63
Norway	Math	−0.28(0.02)***	0.05(0.02)**	0.06(0.07)	0.12(0.08)	0.26(0.07)***	0.16(0.04)***	0.25(0.04)***	0.67	0.59
	Science	−0.26(0.02)***	0.05(0.01)***	0.05(0.04)	0.20(0.05)***	0.2(0.05)***	0.21(0.04)***	0.22(0.04)***	0.71	0.62
Panama	Math	−0.22(0.04)***	0.08(0.02)***	0.07(0.09)	0.14(0.08)+	0.18(0.04)***	0.16(0.06)*	0.25(0.07)***	0.64	0.47
	Science	−0.14(0.03)***	0.05(0.02)**	0.10(0.07)	0.24(0.08)+	0.09(0.06)**	0.26(0.07)***	0.18(0.06)**	0.73	0.57
Peru	Math	−0.28(0.02)***	0.08(0.01)***	0.12(0.07)+	0.14(0.07)+	0.14(0.06)*	0.30(0.05)***	0.10(0.05)*	0.67	0.46
	Science	−0.26(0.02)***	0.06(0.01)***	0.17(0.05)+	0.17(0.08)+	0.10(0.07)*	0.33(0.06)***	0.10(0.05)*	0.72	0.52
Philippines	Math	−0.12(0.04)**	0.01(0.02)	0.26(0.06)***	0.20(0.06)**	−0.03(0.07)	0.17(0.07)**	0.25(0.06)***	0.68	0.53
	Science	−0.24(0.04)***	0.00(0.02)	0.16(0.05)**	0.17(0.06)**	0.13(0.04)	0.27(0.05)***	0.19(0.06)**	0.74	0.59
Poland	Math	−0.27(0.03)***	0.10(0.01)***	0.11(0.06)+	0.08(0.06)	0.23(0.04)***	0.22(0.06)***	0.19(0.06)**	0.66	0.52
	Science	−0.27(0.02)***	0.04(0.01)***	0.06(0.06)+	0.10(0.06)	0.30(0.04)***	0.23(0.05)***	0.21(0.05)***	0.75	0.62
Portugal	Math	−0.28(0.02)***	0.10(0.01)***	0.14(0.04)**	0.08(0.05)	0.21(0.06)***	0.16(0.05)**	0.25(0.05)***	0.70	0.53
	Science	−0.26(0.03)***	0.06(0.01)***	0.05(0.06)**	0.13(0.06)	0.28(0.06)***	0.24(0.05)***	0.18(0.04)***	0.75	0.59
Qatar	Math	−0.25(0.03)***	0.08(0.01)***	0.18(0.03)***	0.12(0.05)**	0.13(0.05)**	0.25(0.04)***	0.18(0.03)***	0.68	0.56
	Science	−0.13(0.03)***	0.03(0.01)**	0.13(0.04)**	0.17(0.04)***	0.14(0.03)***	0.27(0.04)***	0.17(0.03)***	0.72	0.61
Russian Federation	Math	−0.26(0.02)***	0.05(0.01)***	0.08(0.05)	0.09(0.09)	0.23(0.06)***	0.16(0.05)**	0.24(0.05)***	0.61	0.53
	Science	−0.22(0.03)***	0.04(0.01)**	0.10(0.06)	0.12(0.05)	0.22(0.04)***	0.17(0.05)***	0.26(0.05)***	0.69	0.61
Serbia	Math	−0.30(0.03)***	0.07(0.01)***	0.05(0.06)	0.11(0.06)+	0.25(0.06)***	0.18(0.04)***	0.21(0.05)***	0.63	0.54
	Science	−0.23(0.03)***	0.05(0.01)***	0.04(0.04)	0.12(0.08)+	0.27(0.05)***	0.20(0.05)***	0.24(0.03)***	0.69	0.60
Singapore	Math	−0.22(0.02)***	0.09(0.01)***	0.07(0.04)	0.12(0.05)*	0.23(0.05)***	0.25(0.04)***	0.14(0.04)***	0.66	0.51
	Science	−0.22(0.02)***	0.06(0.01)***	0.08(0.03)	0.14(0.05)*	0.24(0.04)***	0.23(0.04)***	0.21(0.04)***	0.77	0.63
Slovak Republic	Math	−0.30(0.03)***	0.11(0.02)***	0.11(0.04)*	0.1(0.06)	0.20(0.05)***	0.22(0.05)***	0.18(0.05)***	0.68	0.47
	Science	−0.22(0.02)***	0.06(0.02)**	0.05(0.04)*	0.2(0.04)***	0.21(0.04)***	0.24(0.04)***	0.18(0.04)***	0.74	0.55
Slovenia	Math	−0.35(0.03)***	0.10(0.02)***	0.08(0.06)	0.15(0.05)**	0.20(0.06)**	0.23(0.05)***	0.15(0.05)**	0.65	0.50
	Science	−0.26(0.03)***	0.05(0.02)**	0.03(0.05)	0.26(0.07)***	0.18(0.05)***	0.21(0.05)***	0.22(0.04)***	0.73	0.60
Spain	Math	−0.28(0.02)***	0.11(0.01)***	0.10(0.03)**	0.08(0.05)	0.22(0.04)***	0.20(0.04)***	0.19(0.03)***	0.61	0.48
	Science	−0.25(0.02)***	0.05(0.01)***	0.07(0.03)**	0.15(0.03)***	0.19(0.04)***	0.20(0.03)***	0.22(0.03)***	0.67	0.56
Sweden	Math	−0.24(0.03)***	0.09(0.01)***	−0.02(0.05)	0.18(0.08)*	0.25(0.06)***	0.22(0.05)***	0.20(0.04)***	0.68	0.55
	Science	−0.19(0.02)***	0.07(0.01)***	−0.01(0.04)	0.21(0.06)*	0.24(0.04)***	0.21(0.05)***	0.21(0.06)***	0.73	0.60
Switzerland	Math	−0.32(0.02)***	0.08(0.02)***	0.05(0.05)	0.17(0.07)*	0.19(0.05)***	0.25(0.05)***	0.16(0.06)**	0.68	0.52
	Science	−0.25(0.02)***	0.06(0.01)***	0.07(0.03)	0.13(0.06)*	0.26(0.05)***	0.25(0.06)***	0.18(0.05)***	0.76	0.60
Thailand	Math	−0.18(0.03)***	0.06(0.02)***	0.06(0.05)	0.23(0.05)***	0.08(0.07)	0.27(0.06)***	0.13(0.06)*	0.56	0.45
	Science	−0.16(0.02)***	0.05(0.02)**	0.11(0.04)	0.19(0.05)***	0.12(0.04)	0.22(0.06)***	0.21(0.06)***	0.68	0.55
United Arab Emirates	Math	−0.33(0.03)***	0.09(0.01)***	0.14(0.04)**	0.18(0.05)**	0.10(0.04)**	0.26(0.03)***	0.15(0.03)***	0.66	0.53
	Science	−0.18(0.03)***	0.06(0.01)***	0.11(0.03)**	0.16(0.03)***	0.18(0.02)***	0.26(0.04)***	0.17(0.04)***	0.73	0.60
Türkiye	Math	−0.3(0.03)***	0.05(0.01)**	0.01(0.05)	0.27(0.05)***	0.14(0.05)**	0.19(0.05)***	0.25(0.05)***	0.68	0.57
	Science	−0.17(0.02)***	0.02(0.01)**	0.01(0.04)	0.29(0.04)***	0.15(0.04)**	0.18(0.05)***	0.28(0.06)***	0.75	0.64
United Kingdom	Math	−0.28(0.02)***	0.09(0.01)***	0.11(0.04)**	0.13(0.05)**	0.17(0.04)***	0.23(0.06)***	0.17(0.05)***	0.64	0.52
	Science	−0.17(0.02)***	0.06(0.01)***	0.06(0.03)**	0.19(0.03)***	0.18(0.04)***	0.15(0.04)***	0.28(0.04)***	0.71	0.61
United States	Math	−0.27(0.02)***	0.1(0.02)***	0.03(0.07)	0.15(0.09)+	0.23(0.06)***	0.26(0.06)***	0.17(0.07)*	0.74	0.57
	Science	−0.2(0.02)***	0.03(0.01)**	0.07(0.04)	0.23(0.06)***	0.16(0.05)**	0.19(0.06)**	0.26(0.07)***	0.77	0.65
Uruguay	Math	−0.27(0.03)***	0.08(0.02)***	0.17(0.07)*	0.07(0.08)	0.17(0.06)**	0.30(0.05)***	0.10(0.05)*	0.66	0.49
	Science	−0.22(0.03)***	0.05(0.02)**	0.10(0.07)*	0.11(0.07)	0.24(0.08)**	0.31(0.06)***	0.11(0.04)*	0.73	0.56
B-S-J-Z (China)	Math	−0.25(0.02)***	0.02(0.01)	0.02(0.06)	0.22(0.04)***	0.19(0.06)**	0.23(0.03)***	0.19(0.05)***	0.66	0.56
	Science	−0.27(0.02)***	0.02(0.01)	0.08(0.03)	0.19(0.04)***	0.18(0.03)***	0.23(0.03)***	0.21(0.04)***	0.74	0.63
Moscow Region (RUS)	Math	−0.29(0.04)***	0.07(0.02)**	0.07(0.08)	0.11(0.09)	0.24(0.07)**	0.12(0.07)+	0.25(0.07)***	0.60	0.55
	Science	−0.22(0.04)***	0.03(0.02)**	0.10(0.08)	0.13(0.07)	0.21(0.08)**	0.17(0.06)+	0.26(0.06)***	0.69	0.66
Tatarstan (RUS)	Math	−0.27(0.03)***	0.04(0.01)**	0.07(0.07)	0.12(0.09)	0.21(0.06)***	0.15(0.05)**	0.25(0.05)***	0.61	0.56
	Science	−0.22(0.03)***	0.02(0.01)**	0.10(0.06)	0.13(0.05)	0.21(0.06)***	0.17(0.04)***	0.26(0.04)***	0.69	0.64
Overall	Math	−0.25(0.00)***	0.08(0.00)***	0.14(0.01)***	0.14(0.01)***	0.15(0.01)***	0.20(0.01)***	0.21(0.01)***	0.71	0.53
	Science	−0.19(0.00)***	0.04(0.00)***	0.09(0.01)***	0.18(0.01)***	0.20(0.01)***	0.22(0.00)***	0.22(0.01)***	0.78	0.62
Overall (Fixed Country Effects)	Math	−0.23(0.00)***	0.07(0.00)***	0.10(0.01)***	0.13(0.01)***	0.17(0.01)***	0.21(0.01)***	0.18(0.01)***	0.75	0.56
	Science	−0.18(0.00)***	0.04(0.00)***	0.07(0.01)***	0.17(0.01)***	0.20(0.01)***	0.23(0.01)***	0.20(0.01)***	0.80	0.63

+*p* < 0.10; *p < 0.05; **p < 0.01; ***p < 0.001. Δ *R*^2^ is the difference between explanatory power of the full model (FEMALE + ESCS + RCLI + RCUN + RCER + RTSN + RTML) and the demographic-only model (FEMALE + ESCS). FEMALE (0 = Male, 1 = Female). Observations were weighted with SENWT. ESCS: index of economic, social and cultural status. RCLI: cognitive process subscale of reading–locate information. RCUN: cognitive process subscale of reading–understand. RCER: cognitive process subscale of reading–evaluate and reflect. RTSN: text structure subscale of reading–single. RTML: text structure subscale of reading–multiple.

In the overall sample with country-fixed effects, an increase in the cognitive constructs employed reading (RCLI, RCUN, and RCER) was associated with an increase in math and science after controlling for gender, socioeconomic status, and text structure subscales of reading. If a student is one standard deviation above the average on all of the RCLI, RCUN, and RCER, most of the increase in math and science will be due to RCER, followed by RCUN, and finally RCLI. In comparison, RCER plays a more significant role in math and science achievement. This makes sense since RCER is a more complex cognitive process than RCUN, and RCUN is a more complex cognitive process than RCLI.

RCER affected math achievement in 58 out of 71 countries and science achievement in 69 out of 71 countries. On average, one standard deviation increase in RCER was associated with 0.17 (*p* < 0.001) and 0.20 (*p* < 0.001) standard deviation increase in math and science. We also found that RCUN affected math achievement in 27 out of 71 countries and science achievement in 55 out of 71 countries. On Average, one standard deviation increase in RCUN was associated with an additional increase of 0.13 (*p* < 0.001) and 0.17 (*p* < 0.001) standard deviation in math and science. Finally, RCLI affected math achievement in 33 out of 71 countries and science achievement in 26 out of 71 countries. On average, one standard deviation increase in RCLI was associated with a further increase of 0.10 (*p* < 0.001) and 0.07 (*p* < 0.001) standard deviation in math and science. In other words, a student one standard deviation above the mean on RCER, RCUN, and RCLI, cumulatively, will have a math score roughly 0.17 + 0.13 + 0.10 = 0.40 standard deviation above an average student in math and 0.20 + 0.17 + 0.07 = 0.44 standard deviation above an average student in science.

An increase in the text structure subscales of reading (RTSN and RTML) was associated with an increase in math and science after controlling for gender, socioeconomic status, and cognitive reading processes. We found that RTSN affected math achievement in 58 out of 71 countries and science achievement in 71 countries. On average, one standard deviation increase in RTSN was associated with 0.21 (*p* < 0.001) and 0.23 (*p* < 0.001) standard deviation increase in math and science. We also found that RTML affected math achievement in 67 out of 71 countries and science achievement in 69 out of 71 countries. On average, one standard deviation increase in RTML was associated with an additional increase of 0.18 (*p* < 0.001) and 0.20 (*p* < 0.001) standard deviation in math and science. In other words, a student with one standard deviation above the mean on RTSN and RTML will have a math score of roughly 0.21 + 0.18 = 0.39 standard deviation above an average student in math and 0.23 + 0.20 = 0.43 standard deviation above an average student in science.

Cognitive constructs employed in reading subscales (RCLI, RCUN, RCER, RTSN, and RTML) explain 56% of the variance in math on average (as low as 0.43 [Tatarstan (RUS)], as high as 63% [Albenia]) and 63% of the variance in science on average (as low as 52% [Tatarstan (RUS)], as high as and 70% [Albenia]). These values are the incremental increase in $R^{2}$ value, in other words, what is left after removing the explanatory power of gender and socioeconomic status. This means, regardless of students’ gender and their socioeconomic background, good readers do substantially better in math and science. While this is still the case, it is still unknown why most of those who are good at math are also good at reading, but vice versa does not hold. Interestingly, adding country-fixed effects does not improve $R^{2}$beyond what is already in the model. This tells us that the conditional mean of math and science across countries (after controlling for gender, socioeconomic status, and subscales of reading) does not vary substantially. This was also confirmed via checking their regression coefficients for fixed effects. This provides evidence against the idea that some cultures are better at math and science. They might be better because they are good readers or come from well-to-do families.

It is not easy to make sense of results by country. Standardized effects and their 95% Confidence Intervals (CI) are reported in Supplement Figures S1, S14. Countries are ordered by their effect size. For math and science subjects, there was a moderate to large negative correlation between the coefficient of RCLI and coefficients of RCUN (*r* = −0.42 and − 0.49, *p* < 0.001) and RCER (*r* = −0.55 and −0.53, *p* < 0.001) across 71 countries. The smaller coefficient of RCLI in a country, the larger coefficients of RCUN and RCER. For example, in math subjects, the Netherlands, Korea, and Latvia were within the bottom five countries on the effect of RCLI, but all three countries are within the top five on the effect of RCER (see Supplement Figures S3, S5). Similarly, in the science subject, Netherlands and Denmark were within the bottom five on the effect of RCLI, but Denmark was within the top five on the effect of RCUN (compare Supplement Figures S10, S11), and the Netherlands was within the top five on the effect of RCER (compare Supplement Figures S10, S12).

For math and science subjects, there was a moderate to a large negative correlation between the coefficient of RCUN and the coefficient of RCER (*r* = −0.49 and −0.43, *p* < 0.001). The larger coefficient of RCUN, the smaller the coefficient on RCER. For example, in math subject, Dominican Republic, Thailand, and Indonesia were within the top five countries on the effect of RCUN, but all three countries were within the bottom five on the effect of RCER (compare Supplement Figures S4, S5). For math subject only, there was a moderate positive correlation between the coefficient of RCUN and the coefficient of RTSN (*r* = 0.30, *p* = 0.01). The larger coefficient of RCUN, the larger the coefficient of RTSN.

Finally, for math and science subject, there was a very large negative correlation between the coefficient of RTSN and the coefficient of RTML (*r* = −0.94 and −0.95, *p* = 0.001). The smaller coefficient of RTSN, the larger the coefficient on RTML. For example, in math subjects, countries Canada and New Zealand were within the bottom five on the effect of RTSN. However, they are both within the top five on the effect of RTML. Similarly, in the science subject, countries Korea, Macao, the United Kingdom, and Denmark were within the bottom five on the effect of RTSN; however, they are all within the top five on the effect of RTML.

## Conclusion and discussion

This study conducted a multi-group path analysis to examine the relationships between cognitive constructs, gender, socioeconomic status, and math and science achievement across 71 countries. The results are as follows: First, increases in the cognitive constructs employed in reading (locating information, understanding, evaluation and reflection, single thinking, and multiple thinking) were associated with increased math and science achievement. “Evaluation and reflection” had the most significant impact, followed by “understanding” and then “locating information.” A student with higher scores on all three cognitive constructs had higher math and science scores. Moreover, “single thinking” and “multiple thinking” substantially impacted math and science scores. Second, the combined effects of cognitive constructs employed in reading explained a significant portion of the variance in math and science scores. On average, they explained 56% of the variance in math and 63% of the variance in science, after accounting for gender and socioeconomic status. Third, adding country-fixed effects did not substantially improve the model’s explanatory power. This suggests that the conditional mean of math and science achievement does not significantly vary across countries when controlling for gender, socioeconomic status, and reading abilities. Finally, there were various inter-country relationships among the coefficients of cognitive constructs employed in reading. For instance, countries with lower “locating information” coefficients often had higher “understanding” and “evaluation and reflection” coefficients. Similarly, countries with lower “single thinking” coefficients tended to have higher “multiple thinking” coefficients.

The current research was one of the first to examine the relationships between five cognitive constructs employed in reading (locating information, understanding, evaluating and reflecting, single and multiple thinking), gender, ESCS, and science and mathematics literacy performances across 71 different countries. Overall findings for 71 countries indicated that “locating information,” “understanding,” “evaluating and reflecting,” “single thinking,” and “multiple thinking,” gender, and ESCS explained the total variance of mathematics 75% and science 80% after controlling for country effects (see Table 3). At the country level, cognitive constructs; locating information, understanding, evaluating and reflecting, and single and multiple thinking explained the total variance ranging from 52 to 70% for science and from 43 to 63% for mathematics across 71 countries (see Table 3). Moreover, ESCI was linked with science and math overall, and across 71 countries except for Brunei Darussalam, Kazakhstan, Philippines, and B-S-J-Z (China). Findings related to cognitive constructs; locating information, understanding, evaluating and reflecting, single and multiple thinking associated with science and mathematics performance for most of the 71 countries. Single and multiple thinking were related to science and mathematics performance for all countries except Costa Rica, in which multiple thinking was not related to mathematics (see Table 3).

This study emphasized the influence of cognitive constructs employed in reading (locating information, understanding, evaluating and reflecting, single and multiple thinking), gender, and ESCS on science and mathematics literacy performance across the 71 countries that highlighted the significance of national cultural context on these relationships. Thus, it was suggested gender, ESCS, “locating information,” “understanding,” “evaluating and reflecting,” “single thinking,” and “multiple thinking” have culturally specific relationships with science and mathematics performance across the 71 countries (see Table 3). As a result, the relationships of these cognitive constructs with science and mathematics performance indicated differences as well as similarities across 71 countries. A follow-up random-effects meta-analysis indicated that effects varied across countries from small to moderate degrees (see Table 4). Among the cognitive constructs examined, understanding appears to be the most consistent across countries, with only small variations observed. In contrast, locating information and evaluating and reflecting show moderate degrees of variation across countries.

**Table 4:** Heterogeneity Estimates.

Predictor	Outcome	*Q*	p	$I^{2}$	$H^{2}$
Female	Math	260.82	0.000	77.47	4.44
	Science	200.08	0.000	68.59	3.18
ESCS	Math	259.07	0.000	75.55	4.09
	Science	182.13	0.000	65.76	2.92
RCLI	Math	100.29	0.010	46.40	1.87
	Science	101.44	0.008	47.54	1.91
RCUN	Math	65.06	0.645	31.22	1.45
	Science	57.77	0.852	29.72	1.42
RCER	Math	100.20	0.010	49.65	1.99
	Science	113.00	0.001	51.26	2.05
RTSN	Math	87.53	0.077	38.11	1.62
	Science	83.10	0.136	38.26	1.62
RTML	Math	104.82	0.004	42.28	1.73
	Science	105.40	0.004	47.35	1.90

Random-effects variance estimates were obtained via Sidik-Jonkman estimator (Sidik and Jonkman, 2005, 2007).

Especially, understanding the relationships among these variables overall and for each country level would provide valuable insights for local and global researchers, policymakers, and educators. Specifically, country-level findings regarding the relationship between cognitive constructs and math and science performance would provide projections for policymakers and educators on which relationships should be improved.

Based on the findings, cognitive constructs; locating information, understanding, evaluating and reflecting, single and multiple thinking were found to be facilitative to science and mathematics literacy for 15 years old students, which was verified by previous studies on the effect of cognition on academic performances (Deary et al., 2007; Hopfenbeck et al., 2018; Roth et al., 2015; Zhang and Bae, 2020). Moreover, locating information, understanding, evaluating and reflecting, single and multiple thinking and academic performance relationships varied substantially across 71 countries, these findings are consistent with the assertion that culture shapes cognition (Markus and Kitayama, 1991). Furthermore, this result was also consistent with Nisbett (2004) proposal, that culture exerts an impact on the development of cognition, and cognitive outcomes, including academic performance.

This study enriches prior research on cognition which has primarily compared a few Western and Eastern cultures and examined only limited cognitive variables. Another critical significance is exploring comprehensive cognitive variables related to academic performances across 71 different countries, which makes it more comprehensive compared to prior studies that only examined limited countries. However, the current study has limitations in analyzing the relationships amongst socioeconomic status, gender, cognitive constructs, and academic performance across 71 countries. The fact that this research contained too many findings from 71 countries makes it impossible to evaluate and discuss these findings at the country level within the scope of the article. In coming research, these relationships could be investigated at the individualism vs. collectivism (Hofstede and Hofstede, 2005) and different cultural levels such as grouped by King et al. (2024) Africa and the Middle East, Confucian, East-Central Europe, East Europe, English Speaking, Latin America, Southeast Asia, West Europe, etc.

At the country level, cognitive constructs demonstrated an interesting reconciliatory pattern with academic performances. There were various inter-country relationships among the coefficients of cognitive constructs employed in reading. For instance, countries with lower “locating information” coefficients often had higher “understanding” and “evaluation and reflection” coefficients. Similarly, countries with lower “single thinking” coefficients tended to have higher “multiple thinking” coefficients. When students search for answers to an item, they use “locating information,” “understanding,” “evaluation and reflection,” “single thinking,” and “multiple thinking” employed in reading at different levels across 71 countries. These findings showed that 15-year-old students used various cognitive constructs at different levels when searching for the right choice across countries. We can see this as an indication that different cognitive paths are used to achieve the same goal across 71 countries. It is a common finding of many studies that cognitive constructs and development differ across cultures (Markus and Kitayama, 1991; Nisbett, 2004). Cultural differences in the cognitive constructs would also lead to differences in the variables associated with these cognitive constructs. Specifically, “locating information,” “understanding,” and “evaluating and reflecting’s” association with academic performance have reflected more apparent cultural differences amongst countries. Furthermore, the minuscule differences in explanatory power between models with and without country-fixed effects tell an interesting story. While the cultural context plays a minor role in predicting outcomes above and beyond SES, gender, and cognitive constructs, the effects of cognitive constructs vary depending on the culture. This pattern suggests a reconciliatory relationship between cultural factors and cognitive constructs in explaining the observed outcomes.

Overall findings for 71 countries indicated that “locating information,” “understanding,” “evaluating and reflecting,” “single thinking,” and “multiple thinking,” gender, and ESCS explained the total variance of mathematics 75% and science 80% after controlling for country effects. On the other hand, cognitive constructs employed in reading explained 71% of the variance in math and 78% of the variance in science. This finding shows that “locating information,” “understanding,” “evaluation and reflection,” “single thinking,” and “multiple thinking” utilized in reading literacy are important determinants of mathematics and science performance and explain majority of the effect of cultural differences on cognitive outcomes. One of the prominent findings of this research is that mathematics and science achievement gaps across countries decrease when controlling country effects. This finding suggests that differences in reading literacy between countries might explain the mathematics and science achievement gap across countries. Science, mathematics, and reading literacy are not independent each of other; performance in science and mathematics is determined by the cognitive constructs employed in reading. Therefore, educational policymakers intending to increase science and mathematics performance should consider cognitive constructs employed in reading literacy. So, policymakers should focus on not only science and mathematics content but also reading literacy to improve math and science performance. Through reading literacy, both cognitive skills could be developed and learned how to use these skills in different contexts. From this point of view, PISA participant countries intending to increase PISA academic performance should give priority to the development of these cognitive constructs; “locating information,” “understanding,” “evaluation and reflection,” “single thinking,” and “multiple thinking.” Country-level education policies on the development of PISA performances should be organized in a way that ensures more effective use of cognitive constructs especially in the fields of reading, science, and mathematics literacy.

To summarize the implications for practice: The first implication, strengthening cognitive constructs; “locating information,” “understanding,” “evaluation and reflection,” “single thinking,” and “multiple thinking” should be a priority in educational programs. Improved cognitive constructs employed in reading not only enhance literacy but also contribute to better performance in math and science literacy. Second, schools can adopt cross-curricular strategies that integrate reading, math, and science education. Fostering connections between these subjects might help students transfer cognitive skills across domains. Third, while country-specific differences exist, the impact of cognitive constructs employed in reading on math and science achievement seems consistent. This suggests that interventions focusing on these cognitive skills could have a broad impact across different cultural contexts. Fourth, further research could investigate the specific mechanisms through which cognitive constructs employed in reading contribute to math and science achievement. Understanding these mechanisms could inform more targeted educational interventions. Last implication, policymakers should consider these findings when designing curriculum frameworks and assessments, ensuring that both reading and cognitive skills are adequately developed to support math and science learning.

In conclusion, the study underscores the importance of cognitive constructs employed in reading; “locating information,” “understanding,” “evaluating and reflecting,” “single thinking,” and “multiple thinking,” gender, and socioeconomic status in explaining math and science achievement across various countries. 15-year-old adolescents’ cognitive constructs positively relate to science and math performances, which implies that using cognitive constructs in school settings is crucial for students’ academic performances. Moreover, as a reflection of country-level relationships, cognitive constructs have correlations with academic performances at different significance levels across 71 countries. These findings provide valuable insights for educators, policymakers, and researchers to design effective interventions and strategies that promote student success in these critical subjects for each of countries.

### Limitations and future directions

Our findings are based solely on the countries included in our dataset, and we do not aim to generalize to all countries that participated in the PISA 2018 cycle. Additionally, we do not claim causality, as we only control for policy-relevant demographic variables at the student level. Future research should investigate causal mechanisms more directly. Future studies could explore how gender and socioeconomic status interact with cognitive constructs in reading to predict math and science achievement. A multilevel response surface analysis may reveal nuanced, multi-faceted interactions, while a person-centered approach could provide insights into the alignment of cognitive constructs and their interrelationships. Finally, these results can be replicated with the PISA 2022 dataset to validate findings and examine potential shifts over time.

## Data Availability

Publicly available datasets were analyzed in this study. This data can be found here: the datasets generated during and/or analyzed during the current study are available in the Program for International Student Assessment repository, https://www.oecd.org/pisa/data/2018database/.
