# Supplementary material for: Understanding the role of cognitive constructs employed in reading in global math and science achievement
Source: Front Psychol. 2024 Nov 25;15:1470977. doi: 10.3389/fpsyg.2024.1470977 (PMC11626531; doi:10.3389/fpsyg.2024.1470977)
Supplement: Supplementary file 1 [file Table_1.docx]

**Supplementary Materials**

**Understanding the Role of Cognitive Constructs Employed in Reading in Global Math and Science Achievement**

Gökhan Arastaman, Metin Bulus, Hakkı Kontaş, & Bahadır Özcan

Figure S1

*Standardized Effect of the FEMALE Variable on MATH by Country (from Table 3)*

*
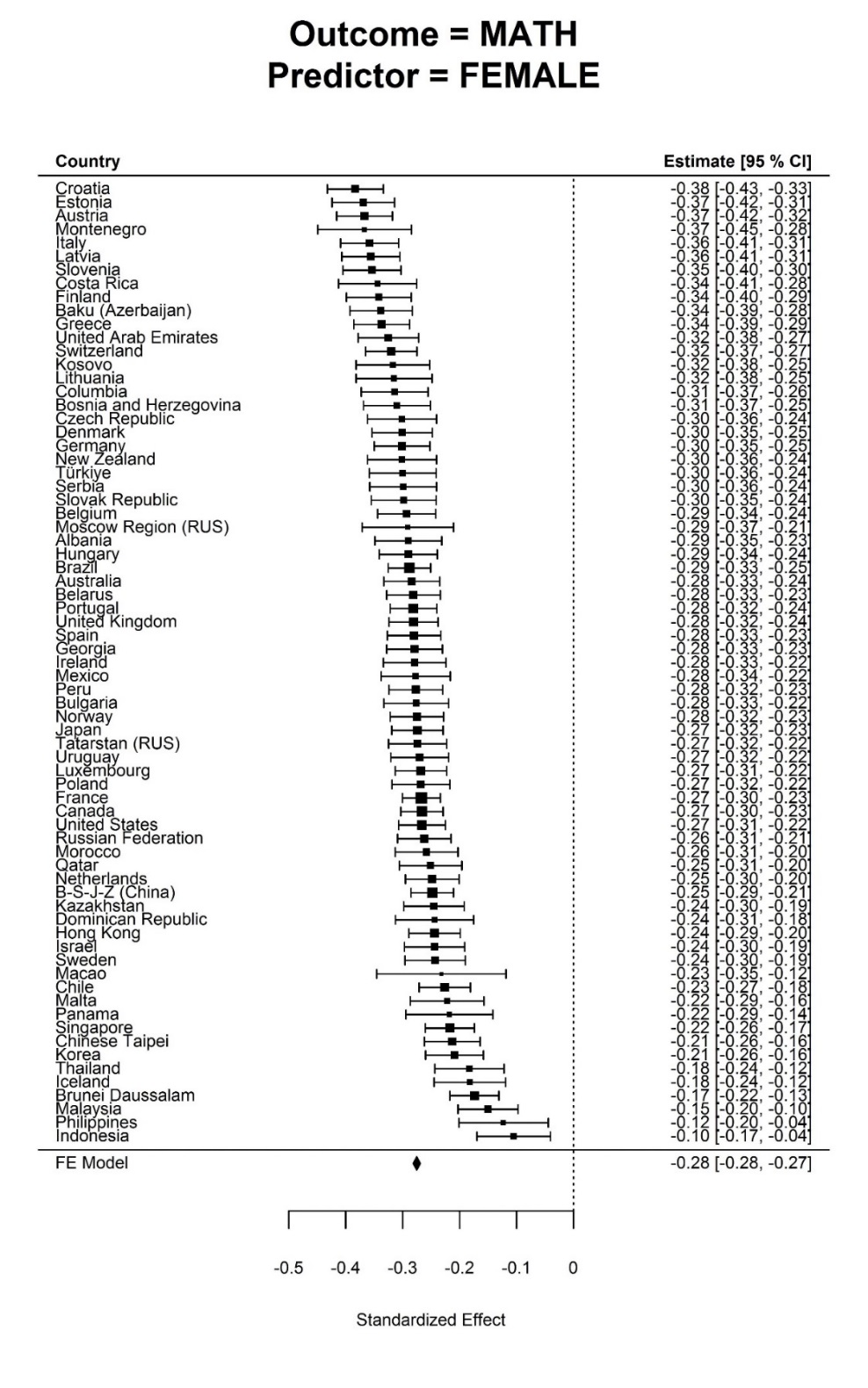
*

Figure S2

*Standardized Effect of the ESCS Variable on MATH by Country (from Table 3)*

*
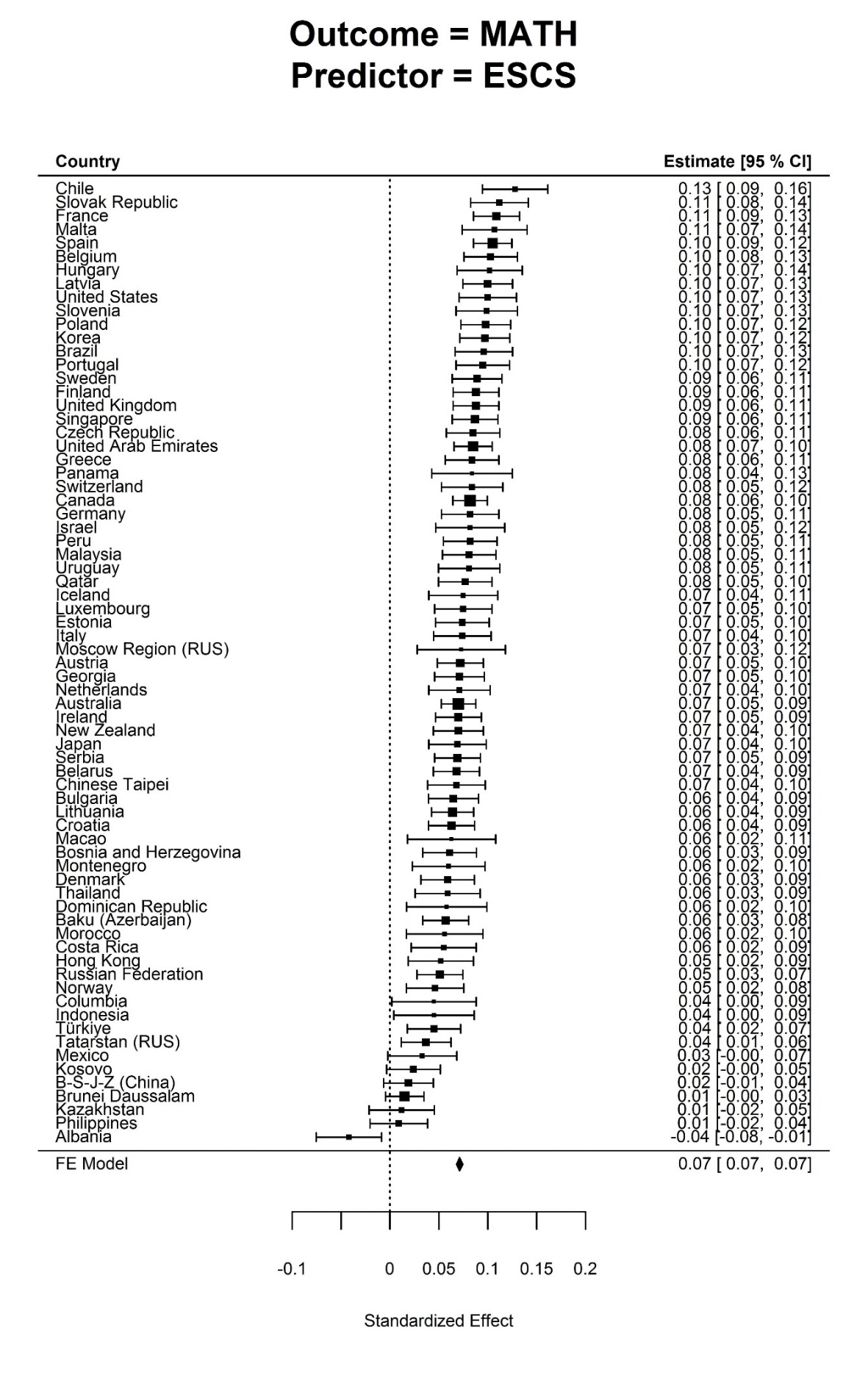
*

Figure S3

*Standardized Effect of the RCLI Variable on MATH by Country (from Table 3)*

*
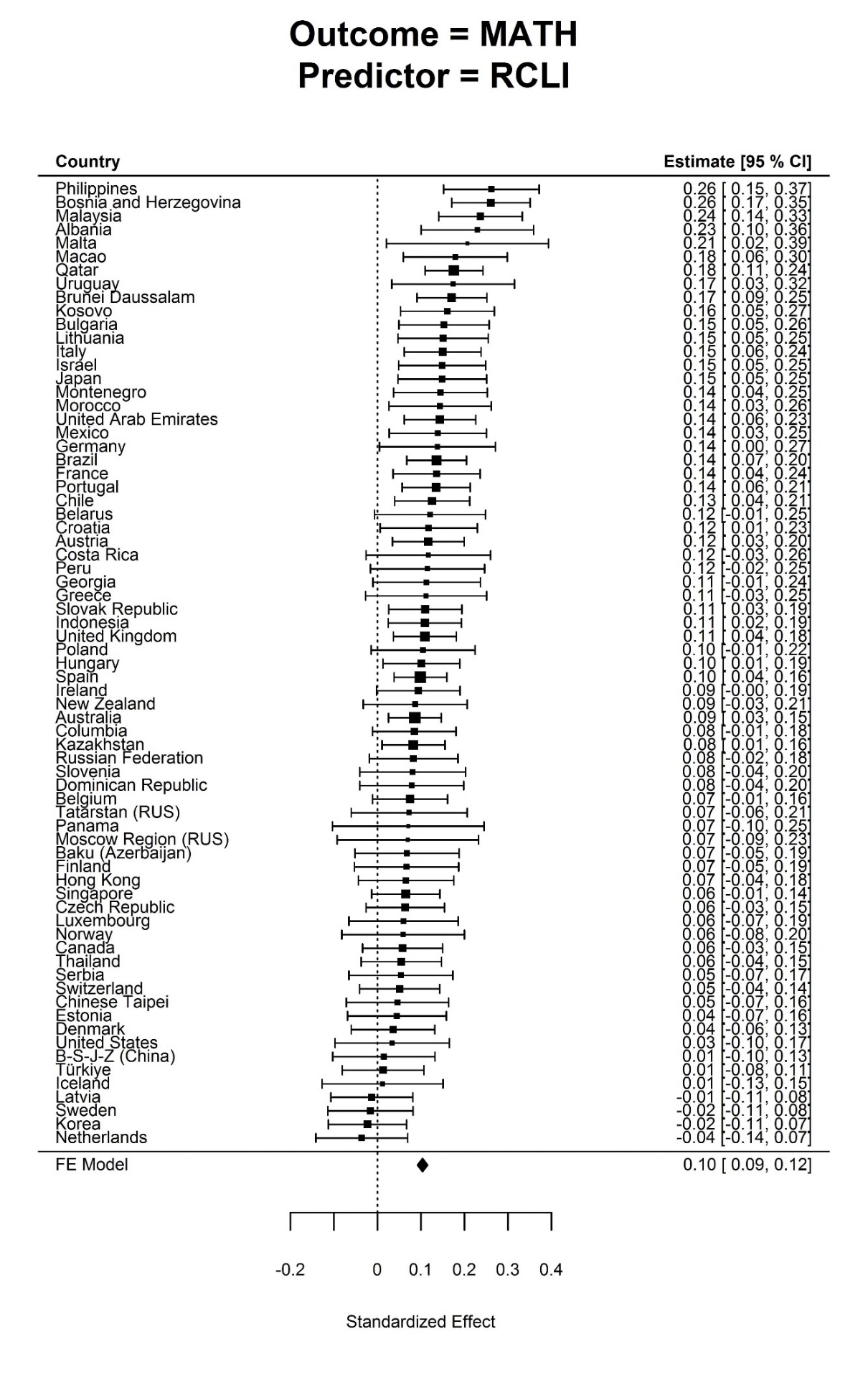
*

Figure S4

*Standardized Effect of the RCUN Variable on MATH by Country (from Table 3)*

*
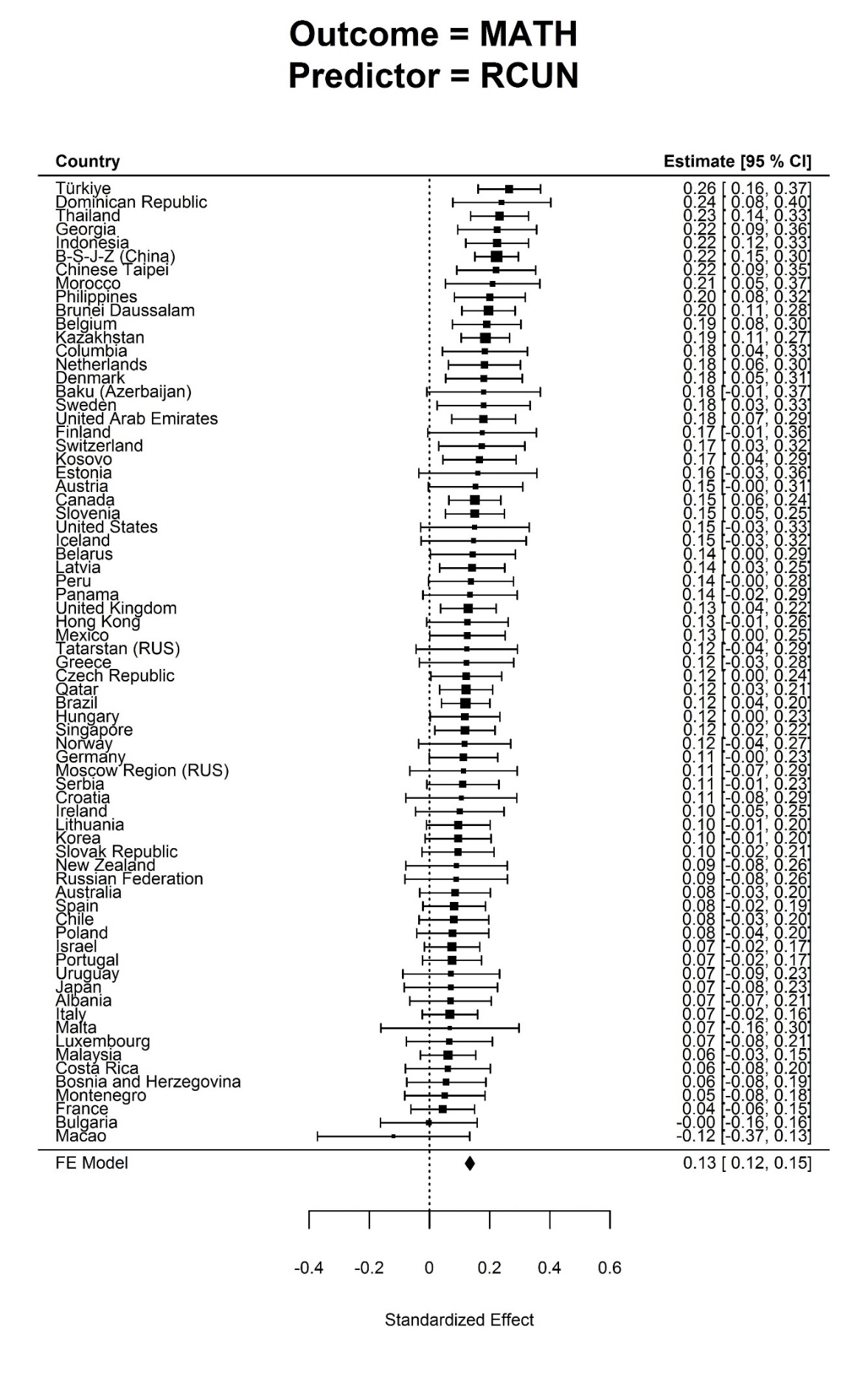
*

Figure S5

*Standardized Effect of the RCER Variable on MATH by Country (from Table 3)*

*
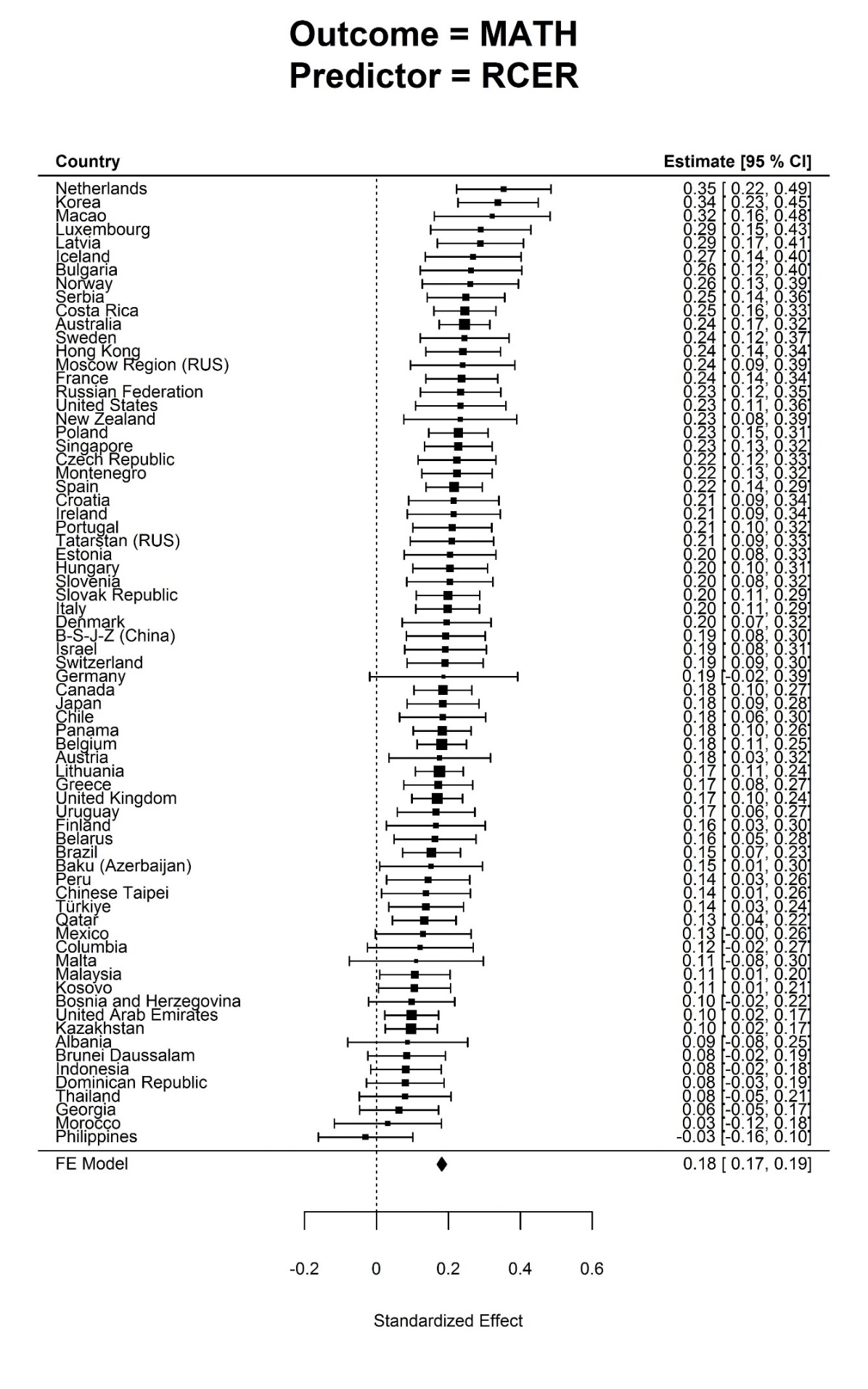
*

Figure S6

*Standardized Effect of the RTSN Variable on MATH by Country (from Table 3)*

*
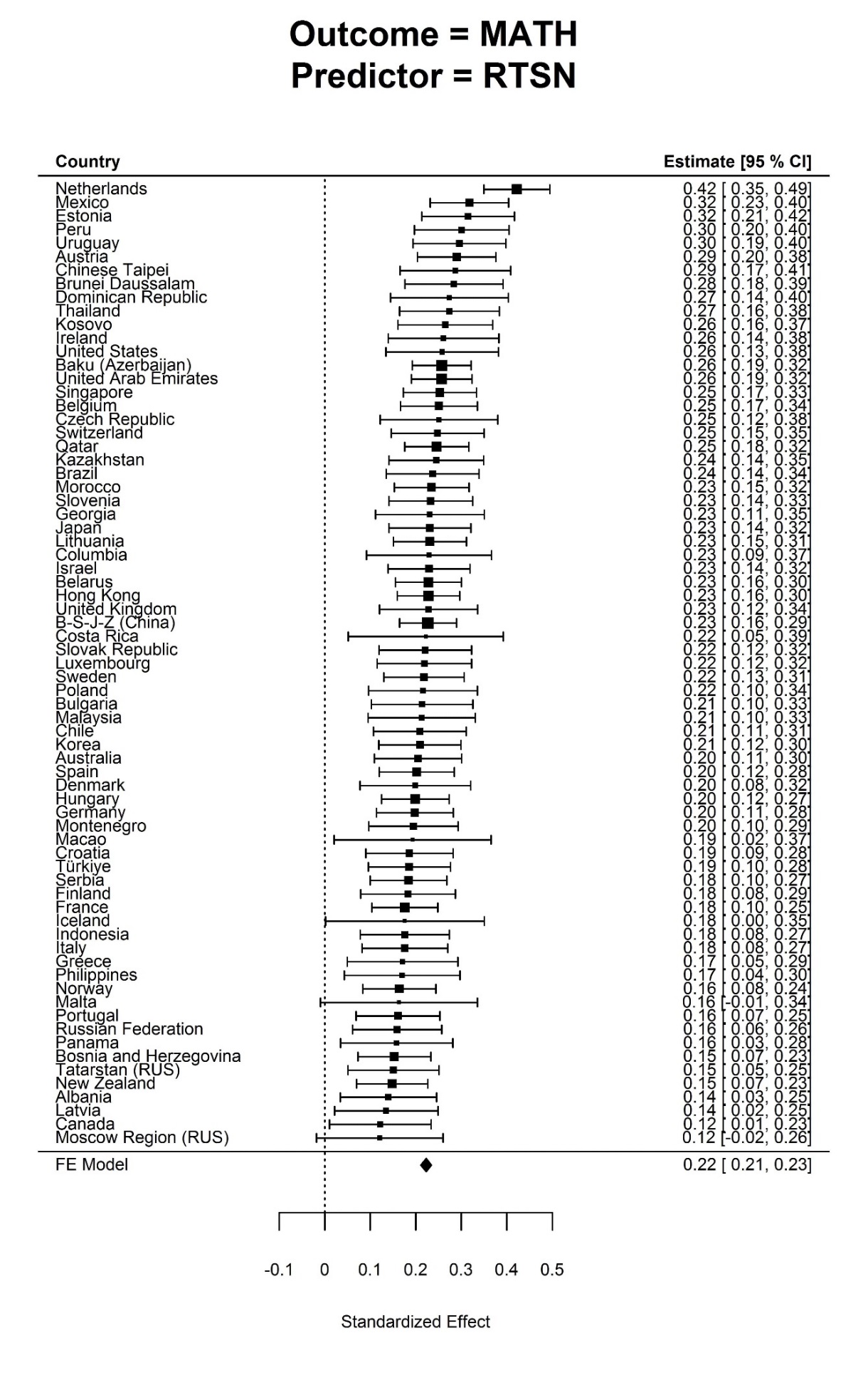
*

Figure S7

*Standardized Effect of the RTML Variable on MATH by Country (from Table 3)*

*
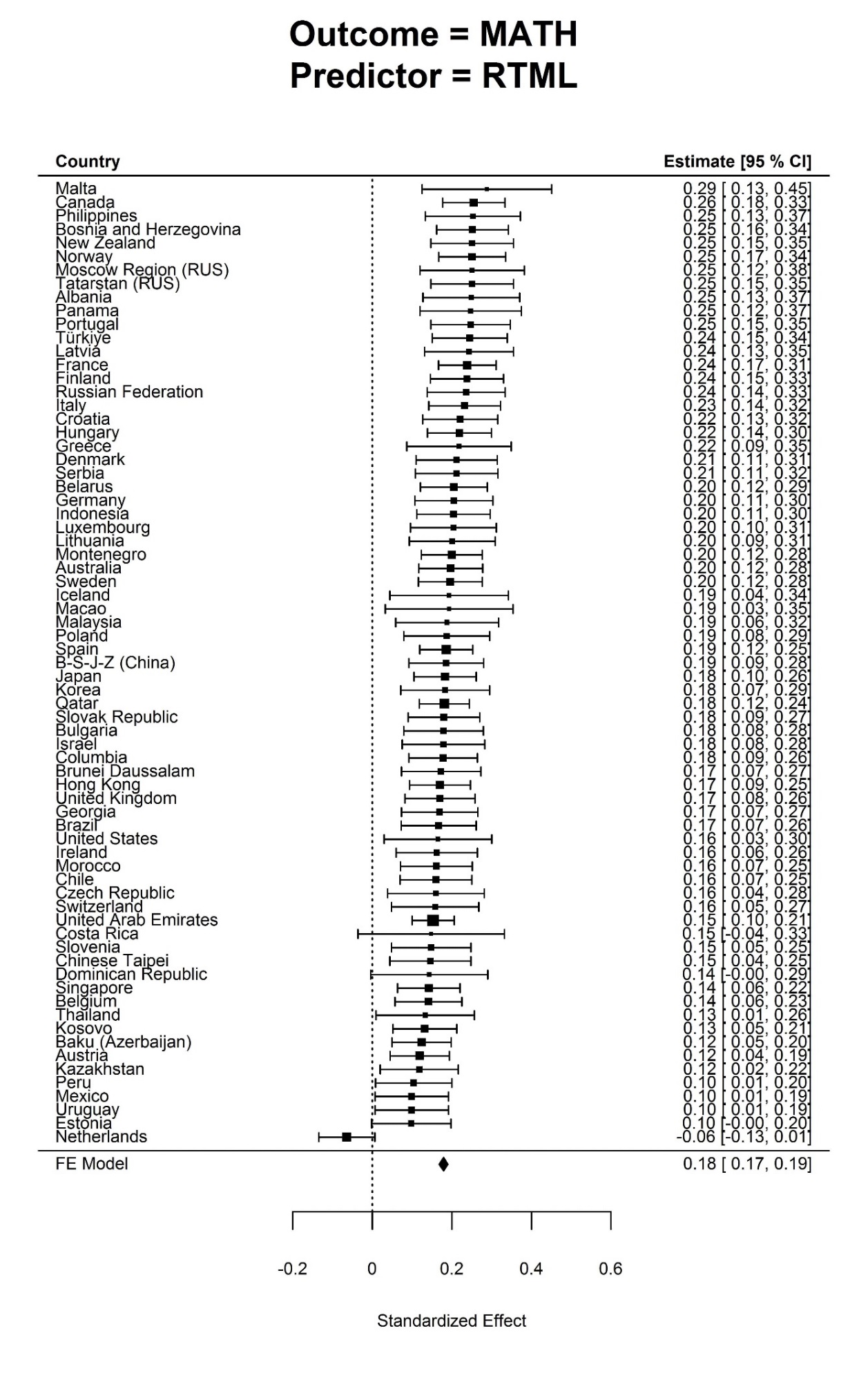
*

Figure S8

*Standardized Effect of the FEMALE Variable on SCIENCE by Country (from Table 3)*

*
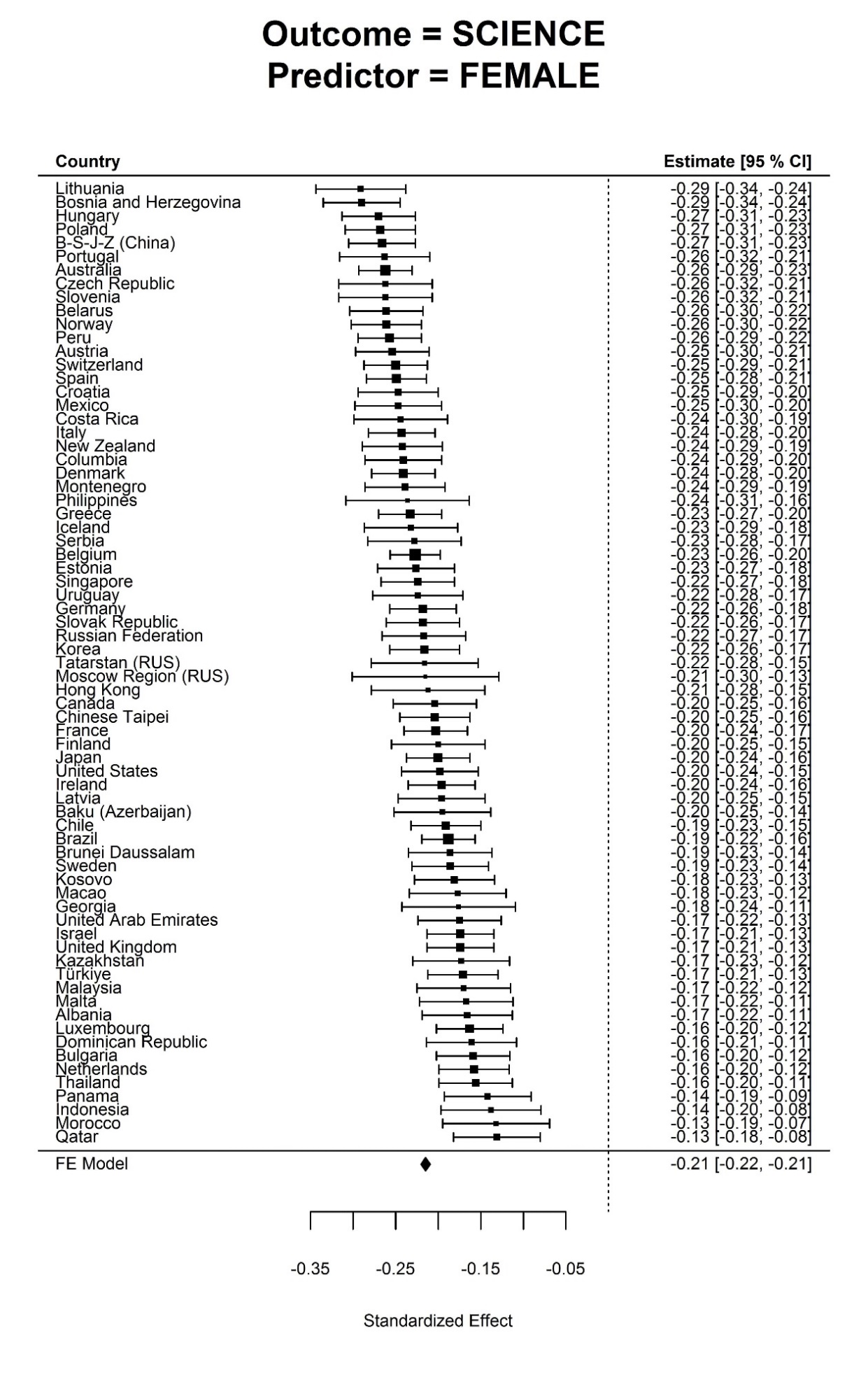
*

Figure S9

*Standardized Effect of the ESCS Variable on SCIENCE by Country (from Table 3)*

*
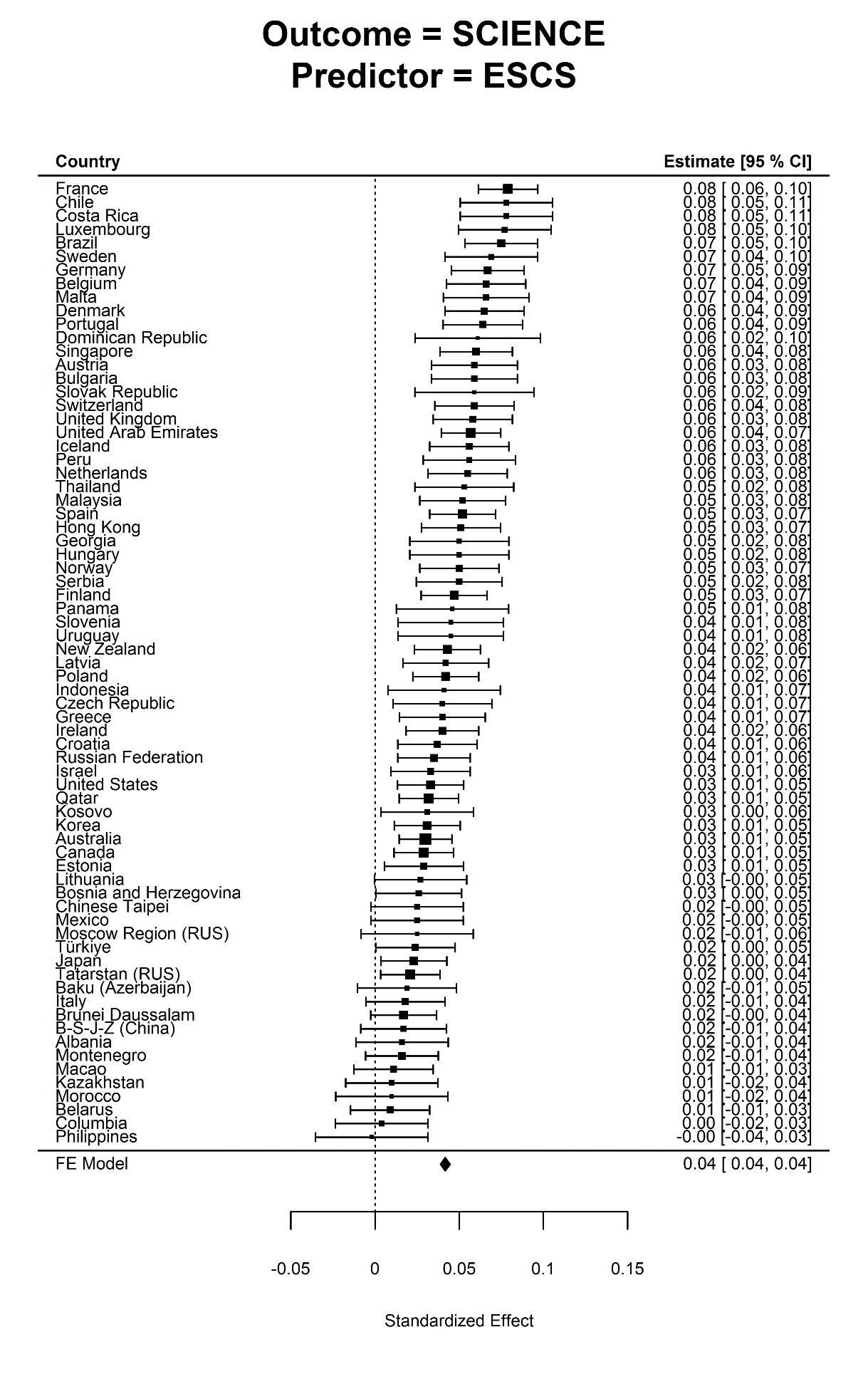
*

Figure S10

*Standardized Effect of the RCLI Variable on SCIENCE by Country (from Table 3)*

*
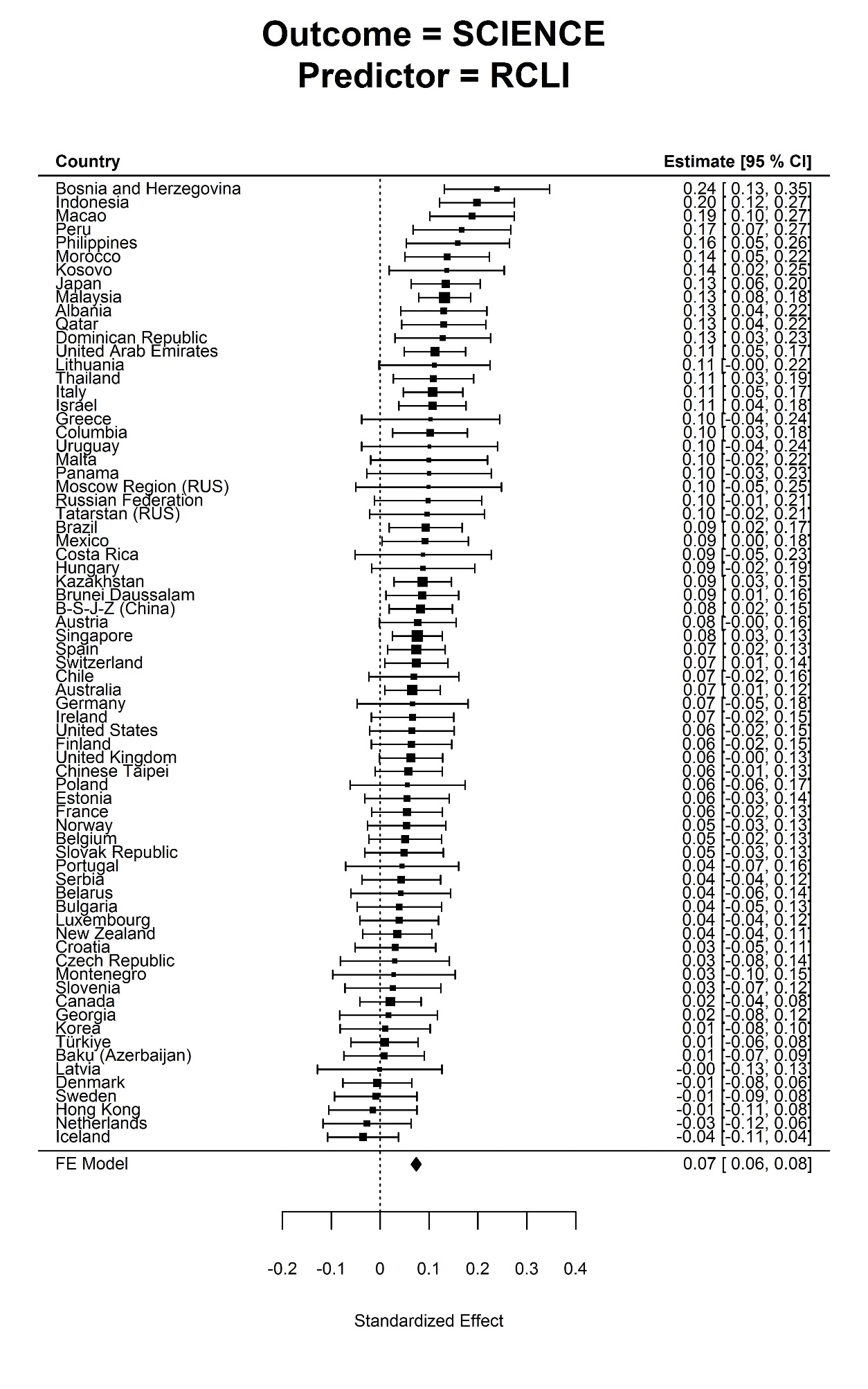
*

Figure S11

*Standardized Effect of the RCUN Variable on SCIENCE by Country (from Table 3)*

*
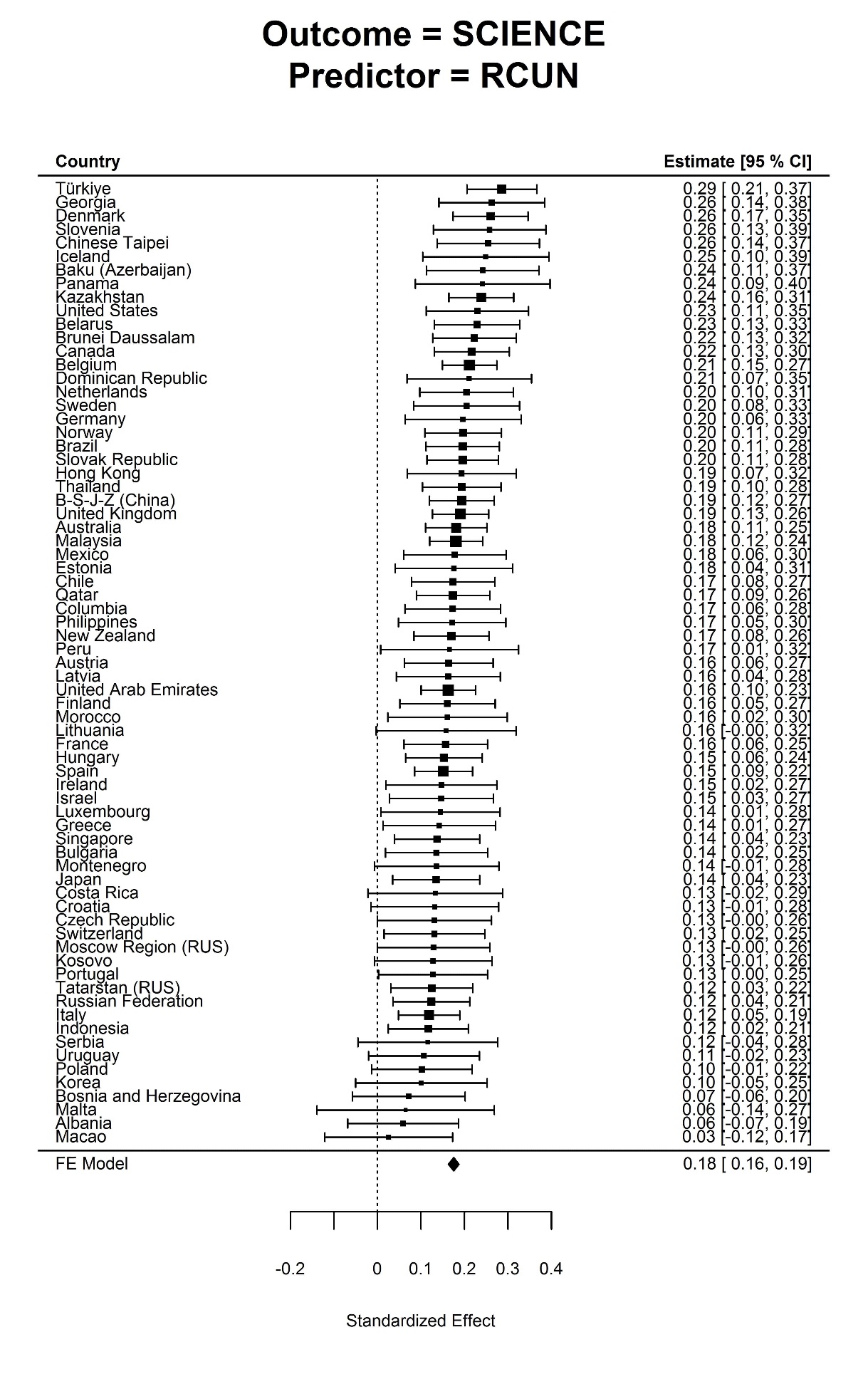
*

Figure S12

*Standardized Effect of the RCER Variable on SCIENCE by Country (from Table 3)*

*
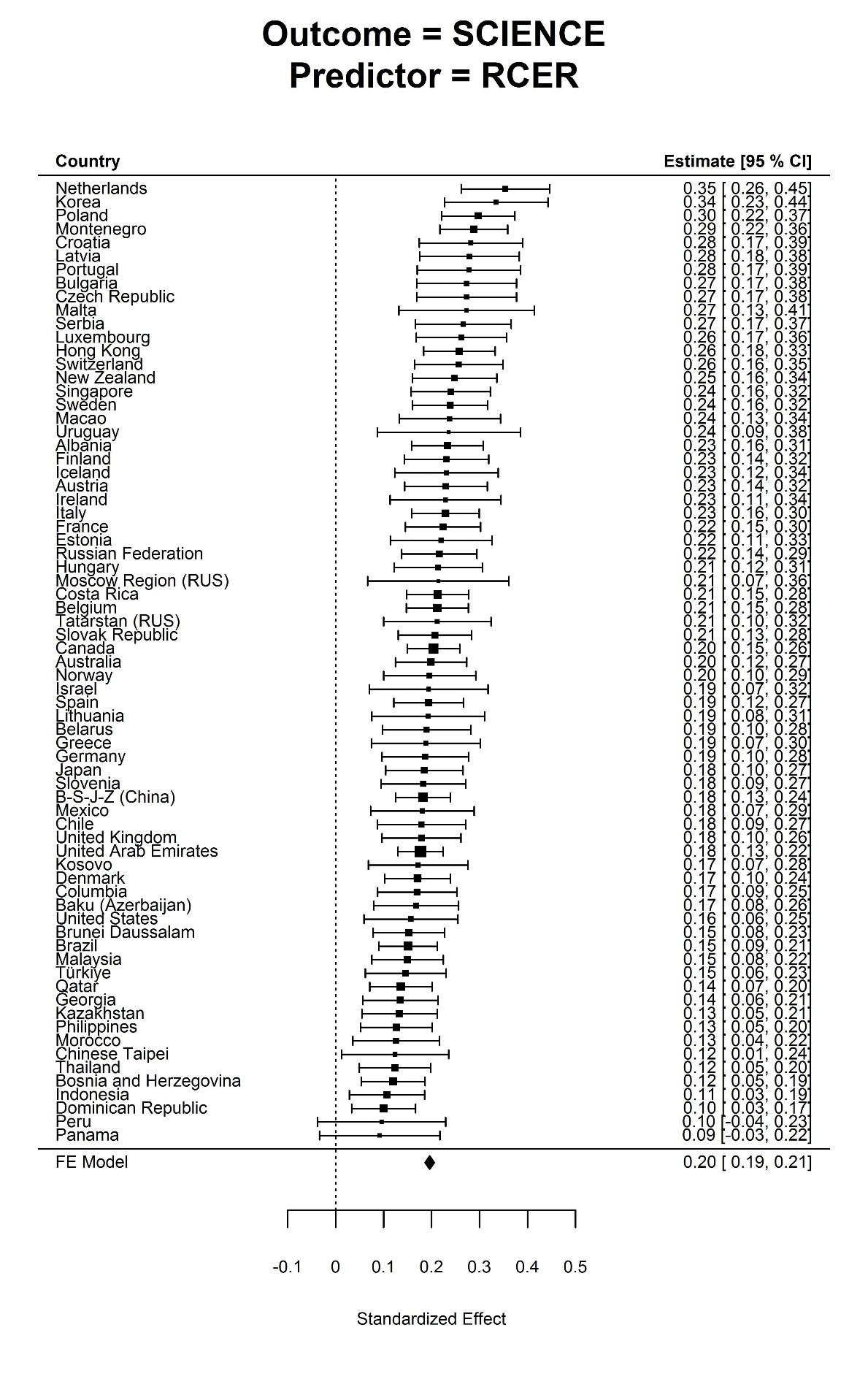
*

Figure S13

*Standardized Effect of the RTSN Variable on SCIENCE by Country (from Table 3)*

*
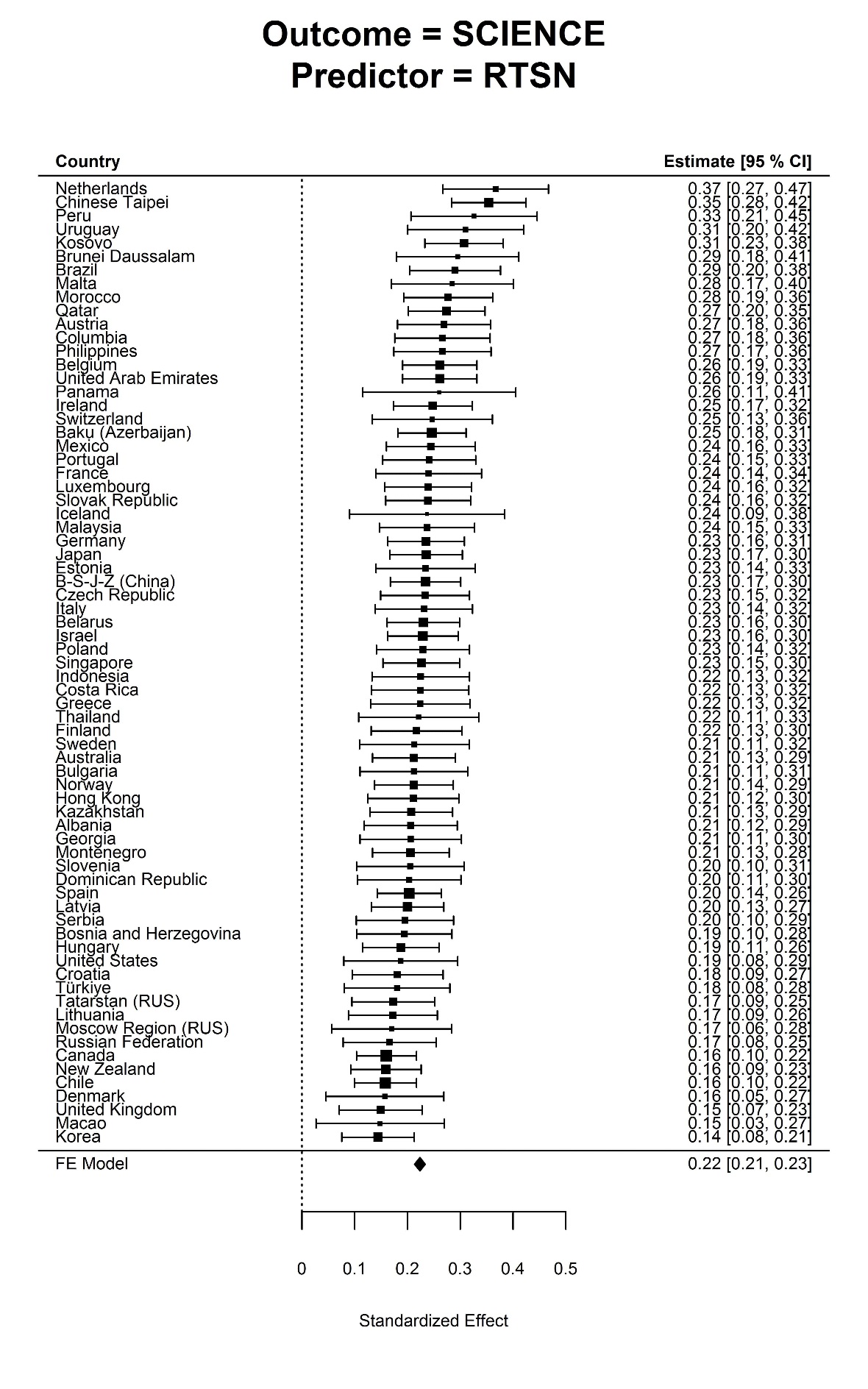
*

Figure S14

*Standardized Effect of the RTML Variable on SCIENCE by Country (from Table 3)*

*
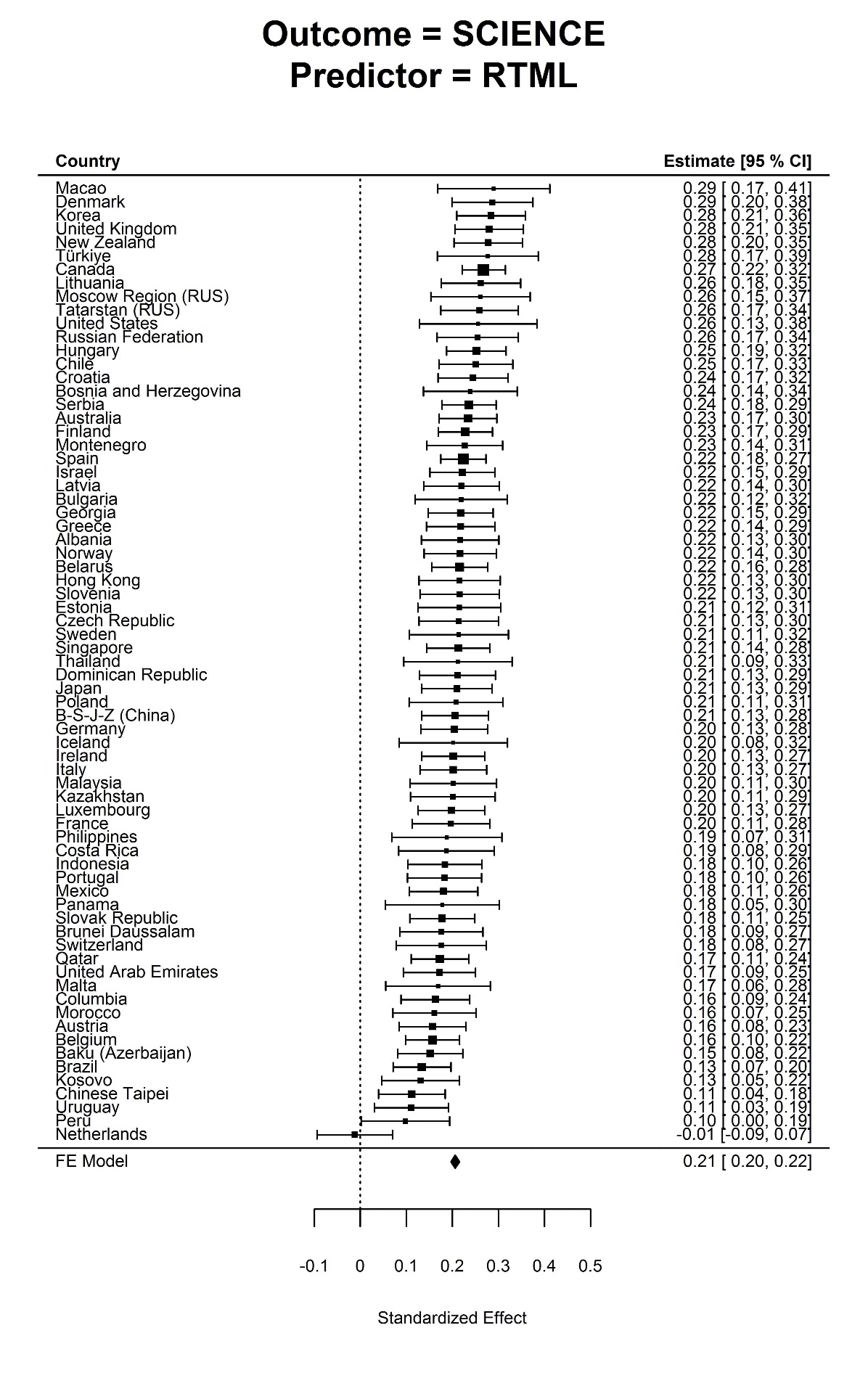
*
